# Synergistic Effect on the Photocatalytic CO_2_ Hydrogenation to Methanol Using Dual Co–Cu Single Atom Poly(heptazine imide): Influence of Pressure on Product Selectivity

**DOI:** 10.1021/acscatal.5c00827

**Published:** 2025-05-21

**Authors:** Alberto García-Baldoví, María Cabrero Antonino, Lu Peng, Liang Tian, Sara Goberna-Ferrón, Germán Sastre, Hermenegildo García, Markus Antonietti, Ana Primo

**Affiliations:** † Instituto de Tecnología Química Universitat Politècnica de València-Consejo Superior de Investigaciones Científicas, Universitat Politècnica de Valencia, Av. De los Naranjos s/n, 46022 Valencia, Spain; ‡ Department of Colloid Chemistry, Max Planck Institute of Colloids and Interfaces, Am Mühlenberg 1, 14476 Potsdam, Germany

**Keywords:** single atom photocatalysis, photocatalytic CO_2_ hydrogenation, pressure effect on methanol formation, poly(heptazine imide) as photocatalyst, dual single atom photocatalyst

## Abstract

Single metal atom-doped materials are gaining importance in photocatalysis since they offer potential maximum atom economy in a system. Herein, the preparation of poly­(heptazine imide) (PHI) carbon nitride materials having Cu^2+^ or Co^2+^ single atom sites or dual Cu^2+^ and Co^2+^ sites is reported. The materials have been characterized by chemical analysis, X-ray diffraction (XRD), and X-ray photoelectron spectroscopy (XPS), while the single-atom nature of the metal dopants is supported by high-resolution high-angle annular dark-field scanning transmission electron microscopy (HAADF-STEM) and X-ray absorption spectroscopy (XAS). The latter also shows a pronounced Cu^2+^–Co^2+^ coordination. The resulting three metal-PHI samples were then explored as photocatalysts for the photocatalytic activation of CO_2_ reduction at various pressures from ambient to 35 bar. A drastic change in the products from CO and CH_4_ under ambient pressure to formic acid and methanol at high pressure was observed, with formic acid being the predominant product at intermediate pressures. The products derived from CO_2_ were firmly confirmed by ^13^C isotopic labeling monitored by gas chromatography-mass spectrometry (GC-MS) (gas products) or ^1^H NMR spectroscopy (liquid products). A synergy between Cu^2+^ and Co^2+^ was observed in the photocatalytic experiments, the activity following the order Co–Cu/PHI > Cu/PHI > Co/PHI and interpreted as derived from the complementary action of each cation, Cu promoting H_2_ activation better than Co and Co promoting hydrogenation of adsorbed CO at lower energy than Cu. These findings show the potential of synergistic effects among different single atoms on a semiconducting support to enhance photocatalytic activity. In addition, the data through light on the importance of pressure to control the product distribution in the photocatalytic CO_2_ hydrogenation toward the more valuable liquid products.

## Graphical abstract

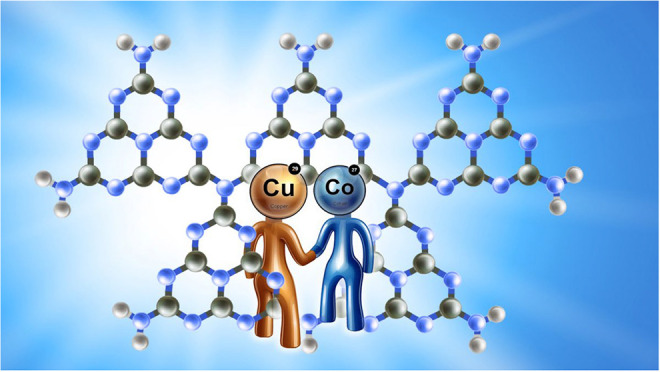

## Introduction

There is an increasing interest in extending the research of single atom metal catalysis from thermal and electrocatalysis to photocatalysis.^1^ Single atoms of metal can enhance the efficiency in a photocatalytic reaction in different ways, including by increasing the charge separation efficiency, by single atoms acting as shallow charge carrier trapping sites and by providing well-defined catalytic sites that after the electron transfer can promote subsequent dark steps with an adsorbed substrate.^2^

While the activity of one metal element as a single atom has been scarcely reported in photocatalysis,^3^ even less documented is the contribution to the photocatalytic activity of the combination of two metals deposited as single atoms in the same catalysts, representing a vast potential in exploiting the synergistic effect among single atoms of two or more metals. This is the step to analyze cooperation or synergisms between different metals in different steps of the mechanism to increase the (photo)­catalytic activity. Graphitic carbon nitride has been one of the favorite single atom supports to be used in photocatalysis, since these materials are active semiconductors with appropriate band positions, while providing well-defined nesting sites on the polyazine framework.^4,5^

Photocatalytic reduction appeared as one of the most appealing reactions to convert CO_2_ into fuels and chemicals, particularly when using natural solar or visible light as the excitation source.^6^ Although H_2_O is the most attractive source of electrons and protons for photocatalytic CO_2_ reduction (as in natural photosynthesis), the efficiency of the reaction is still very low due to the unfavorable endergonicity of the reaction. Besides thermodynamics, the kinetics of photocatalytic CO_2_ reduction is also slow, on the one hand as a consequence of the large number of photons, electrons, and protons involved in the process. In order to have a photocatalytic CO_2_ reduction that can be compared with thermal catalysis (and to be able to analyze all intermediate states), H_2_ is used as an electron and H^+^ donor agent, provided that CO_2_ conversion, product selectivity, and operation conditions of the photoassisted catalytic process are more favorable than those of the thermal reactions.^7,8^ Considering atom economy and possible catalytic enhancement, single metal photocatalysts are, to our opinion, the best candidates to bring photocatalysis closer to the requirements for commercial application.

Synthetic photocatalytic CO_2_ hydrogenations are commonly carried out at ambient pressure, maybe preadjusted in the mind by the example of natural photosynthesis.^7^ In contrast, thermal catalytic CO_2_ hydrogenations require considerable pressure to occur.^9^ Inspired by this obvious dialectics, we would like to establish what the influence of pressure on artificial photocatalytic CO_2_ hydrogenation is. Interestingly, this parameter has remained mostly ignored in the field.

Herein, a double metal single atom (Cu and Co) poly­(heptazine imide) (PHI) is prepared and used as a photocatalyst for CO_2_ hydrogenation. Cu is here a typical CO_2_ reduction site, while Co is an archetypical choice for the necessarily co-occurring oxidation process or the hole activation site. A comparison with the performance of Cu or Co single atoms shows a synergy of the double single atom catalyst, a fact that has been then rationalized by density functional theory (DFT) calculations as derived from the superior H_2_ activation of Cu in comparison to Co and the superior CO hydrogenation ability of Co in comparison to Cu. Besides the synergistic effect, we also report a strong pressure dependence of the product distribution. In contrast to the reaction at atmospheric pressure resulting in CO and some CH_4_, a pressure of 35 bar shifted the product distribution to formic acid and methanol. This product selectivity change indicates that, as in thermal catalysis, pressure also has a dramatic influence on photocatalytic reactions, a fact that has been almost ignored so far.

## Results and Discussion

### Photocatalyst Preparation and Characterization

M/PHI samples were prepared starting from 5-aminotetrazole that is polycondensed in a molten salt flux constituted of a eutectic mixture of LiCl/KCl containing minor amounts of CuCl_2_ or CoCl_2_ or a mixture of both at 550 °C. At this reaction temperature PHI is formed from the tetrazole precursor, while Cu^2+^ or Co^2+^ or both become incorporated into the material that otherwise contains only K^+^ as the charge-balancing cation of the imide ions. Due to its small size, Li+ ions do not become incorporated in the final material.^10^ The materials are denoted as M/PHI in which M indicates the transition metal present in the structure depending on the synthesis. Cu^2+^ and Co^2+^ were selected as single atom sites based on their known activity to promote thermal catalytic CO_2_ hydrogenation.^11^ Note that all of these M/PHI samples contain simultaneously also K^+^ from the molten salt to compensate for the further imide negative charges of PHI. Scheme 1 illustrates the synthesis process and the ideal structure of the M/PHI materials.

**Scheme 1:**
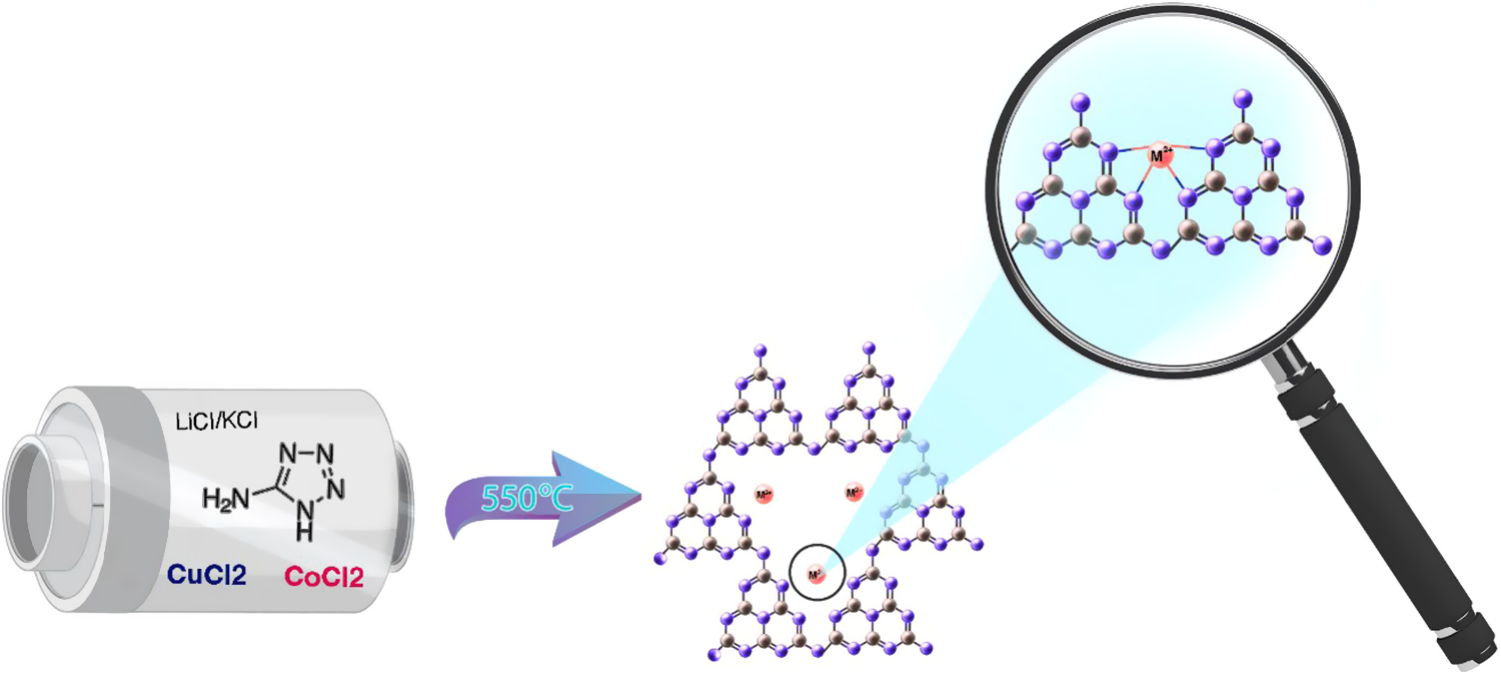
Precursors, Synthetic Route, and Structure of M/PHI Used as Photocatalysts^a^

a The three metal sites are possible, not necessarily taken at the same time. In case of favorable interactions, they however set the base of multi-metal (up the three) co-localization in one pore.

Chemical analysis revealed that the metal content in Cu/PHI and Co/PHI was 3.0 and 0.6 wt % for Cu and Co, respectively. In the case of Co–Cu/PHI the Cu and Co contents were 1.8 and 0.9 wt %, respectively. It should be noted that the synthetic procedure based on the simple mixing of four salts in the flux makes it difficult to predict beforehand the exact metal loading that finally will contain the resulting M/PHI solids. This is due to the fact that the molten flux contains an excess of KCl in comparison to MCl_2_ and incorporation of M^2+^ is a process occurring concertedly with PHI framework formation. Although this different metal loading can play a role in the photocatalytic activity, it should be noted that the total metal loading in Cu/PHI and Co–Cu/PHI is similar, differing only by 0.3 wt % of total metal content. Table 1 summarizes the materials under study and their relevant atomic compositions determined by elemental analysis of the resulting M/PHI samples.

**Table 1:** Photocatalysts Used in This Study and Their Cu and Co Content

catalyst	Cu (wt %)^a^	Co (wt %)^a^
Cu/PHI	3.0	-
Co/PHI	-	0.6
Co–Cu/PHI	1.8	0.9

a Determined by ICP-OES elemental analysis.

Formation of PHI scaffold was confirmed by powder X-ray diffraction (XRD), ^13^C NMR, vibrational spectroscopy, and diffuse reflectance ultraviolet–visible (UV–vis) absorption spectroscopy (Figures S1, S2, S3, and S4 in Supporting Information). The oxidation state and information about the coordination environment in Co–Cu/PHI were provided by X-ray photoelectron spectroscopy (XPS) analysis in which the corresponding elements, C, N, and O, were detected (Figure 1). With this technique, the metals Cu and Co could not be detected due to their low concentration. It may also happen that Cu and Co proportion on the PHI external surface is even lower than the average, due for instance to the fact that washings to remove the excess of chloride salts depletes also from outermost Cu or Co. Deconvolution of the experimental high-resolution XPS peaks revealed the presence of three, wo, and three components for C, N, and O respectively. In the case of C 1s signal (Figure 1a) the three contributions at 284.6, 286.7, and 288.3 eV correspond to the C–C bond, surface C–OH and CN_3_ carbon atoms in the heterocyclic ring, respectively.^12^ The peaks at 293 and 295.7 eV correspond to satellites. The N 1s signal (Figure 1b) can be deconvoluted into two peaks, with the binding energies at 398.6 and 400.7 eV assigned to NC_2_ atoms in the heterocycle ring plus NC_3_ atoms in the center of the ring, and nitrogen atoms of −NH, –NH_2_ groups connecting the triazine rings, respectively. The binding energy values and their relative proportions for the C 1s and N 1s components are in accordance with the PHI structure as previously reported.^13^ The O 1s spectrum (Figure 1c) is composed of three peaks of deprotonated O^–^ atoms at 528.8 eV, the main contribution of surface hydroxyl groups C–OH at 531.4 eV and surface adsorbed water at 533 eV. Supporting Information presents the deconvolution of XPS peaks of Cu/PHI (Figure S5) and Co/PHI (Figure S6). The two-dimensional (2D) morphology of M/PHI was determined by scanning electron microscopy (SEM) in which the expected flake characteristics of K-PHI were observed. Aberration-corrected, high-angle annular dark-field scanning transmission electron microscopy (AC-HAADF-STEM) provides a definitive confirmation of the presence of single atoms, both for Cu^2+^ and Co^2+^ in the PHI support. Figure 2 includes representative images of AC-HAADF-STEM to illustrate the single atom structure of the M/PHI. AC-HAADF-STEM images show conclusively the single-atom nature of the transition metal on the PHI structure. In these images, the white dots on a black background indicate the presence of Cu (Figure 2a,2b) or Co (Figure 2c,2d). At this magnification with *quasi*-atomic resolution, no aggregates can be observed. Figure 2d even shows some details of the PHI framework. Also for Co–Cu/PHI, the single atom distribution can be deduced.

**Figure 1:**
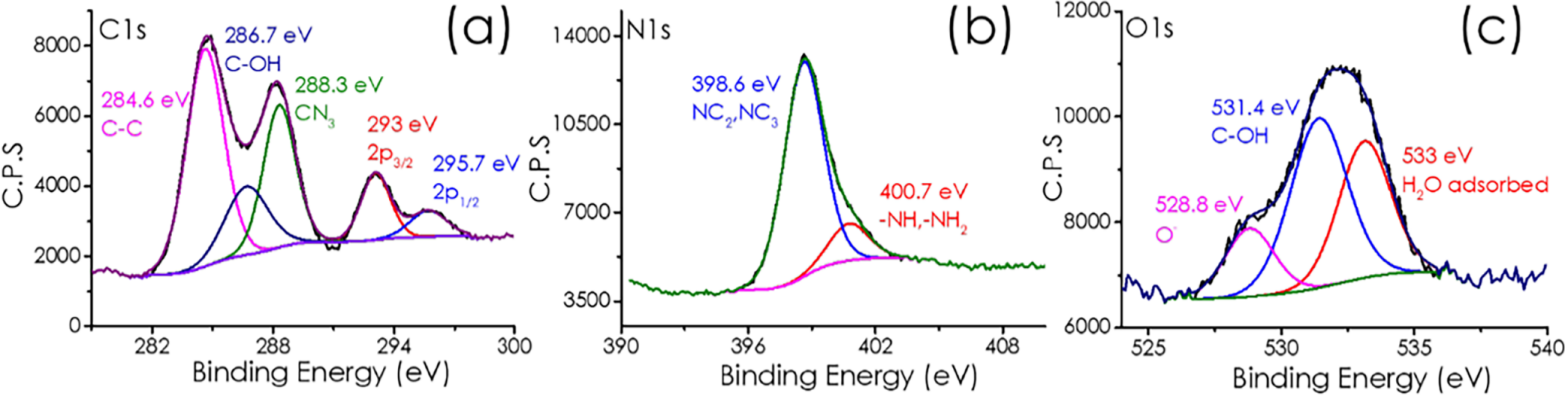
XPS spectral analysis of sample Co–Cu/PHI: (a) C 1s, (b) N 1s, and (c) O 1s.

**Figure 2:**
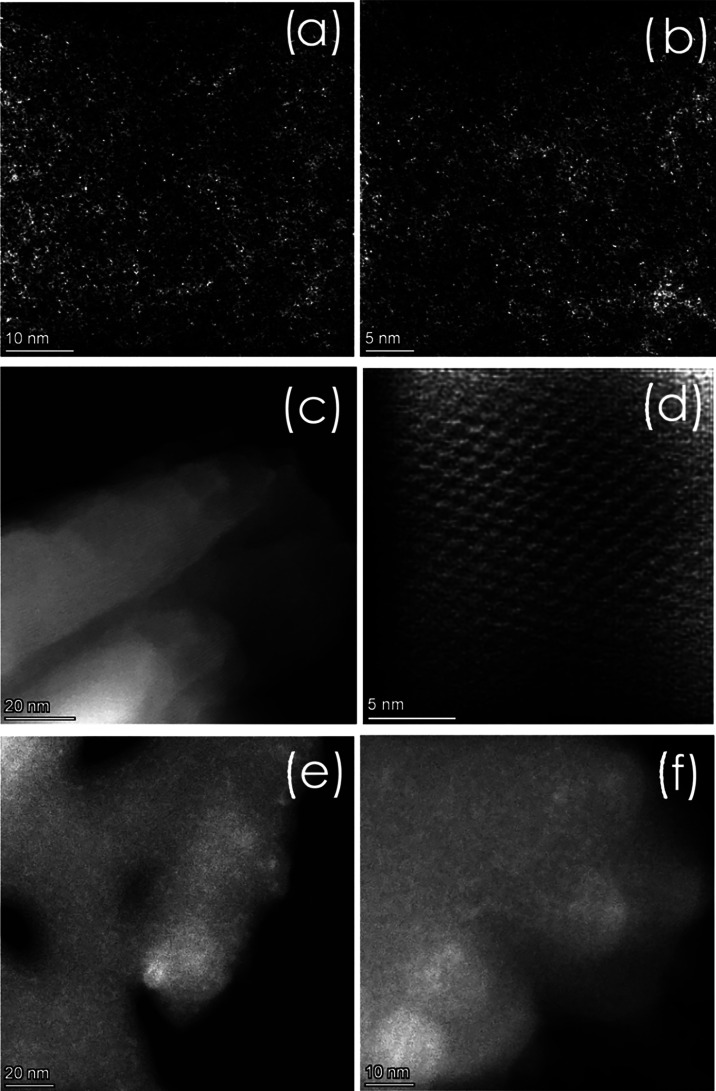
AC-HAADF-STEM images of (a, b) Cu/PHI; (c, d) Co/PHI; and (e, f) Co–Cu/PHI.

The presence of metal single atoms on M/PHI was also supported by Cu and Co K-edge X-ray absorption spectroscopy (XAS). The X-ray absorption near-edge structure (XANES) absorption edge offset can be used to evaluate the average oxidation state of the metal. Figure 3a shows the Cu K-edge XANES plot and comparative analysis with the test results of Cu foil, CuO, Cu_2_O, and CuPc (Pc: phthalocyanine). As shown in the dotted box of Figure 3a, the absorption edge of Cu/PHI and Co–Cu/PHI samples was located between Cu_2_O and CuO, indicating that the core electron density of Cu is between +1 and +2. Interestingly, the absorption edge position for Cu on Co–Cu/PHI is shifted to the right compared to Cu/PHI, indicating a lower electron density of Cu closer to +2. Figure 3c shows the Co K-edge XANES spectra and comparative analysis with the test results for Co foil, CoO, Co_3_O_4_, and CoPc. As shown in the dotted box of Figure 3c, the absorption edge position of Co/PHI and Co–Cu/PHI samples is quite close to that of CoO, indicating that the valence of Co is about +2.

**Figure 3:**
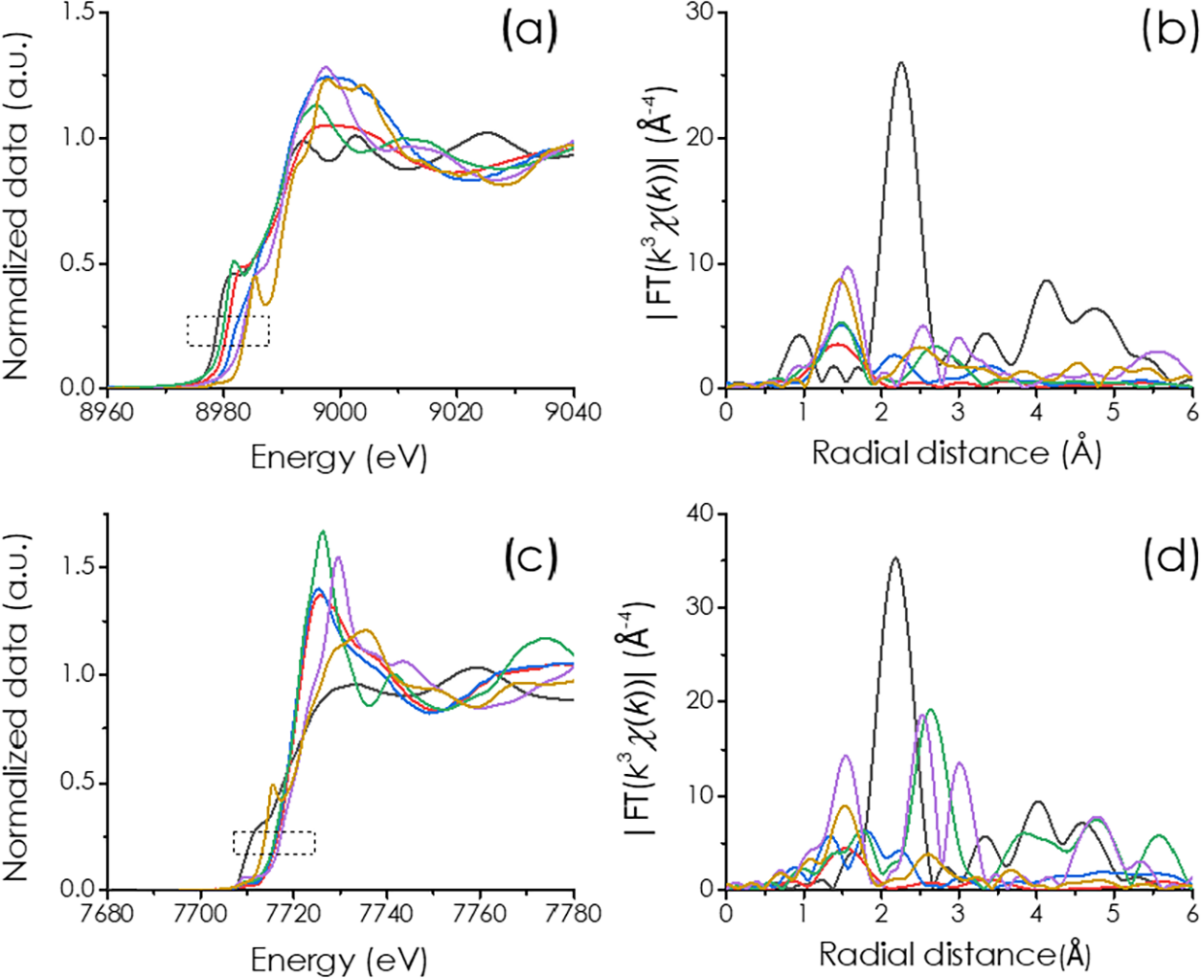
(a) Cu K-edge XANES and (b) Cu K-edge R-space EXAFS spectra of Co–Cu/PHI (blue) and Cu/PHI (red) with references of Cu foil (black) and CuO (purple), Cu_2_O (green) and CuPc (khaki). (c) Co K-edge XANES and (d) Co K-edge *R*-space EXAFS spectra of Co–Cu/PHI (blue line) and Co/PHI (red) with references of Co foil (black) and CoO (green) and Co_3_O_4_ (purple) and CoPc (khaki).

Figure 3b,3d show the Fourier transforms of the EXAFS plots of Co and Cu in PHI and their corresponding reference compounds. EXAFS fitting curve and fitting results are shown in Figures S7–S8 and Supporting Information Table S1. The Fourier transform of EXAFS data of Cu/PHI and Co–Cu/PHI (Figure 3b) shows the major scattering peak centered at ≈1.4 Å, corresponding to the Cu–N bonds in the first coordination sphere. Sample Cu/PHI does not show the longer-range Cu–Cu scattering path at 2.5 Å, implying that the sample does not have a significant proportion of Cu clusters. However, sample Co–Cu/PHI shows a second peak at 2.18 Å that indicates a Co–Cu interaction. The Fourier transform of EXAFS spectra of Co (Figure 3d) displays one strong peak at around 1.5 Å for Co/PHI, which is mainly ascribed to the Co–N coordination at the first shell. The minor peak around 2.5 Å can arise from a second Co–N–C coordination shell (∼2.60 Å for CoPc reference). Interestingly, for the Co–Cu/PHI sample the spectrum shows the split of the first Co–N coordination shell in two groups of distances. While the feature of the Co–Co bond (∼2.2 Å) observed for Co foil is absent in Co/PHI, the Co–Cu/PHI sample shows the appearance of a peak at ≈2.2 Å that indicates a Co–Cu neighborhood.

The Co K-edge EXAFS fitting curve and fitting results of Co are shown in Figure S7 and Table S1. The Co-PHI sample conforms to a Co–N–C type single atom structure, with Co–N and Co–N–C coordination numbers of 4.2 and 2.3, respectively. For the Co–Cu/PHI sample, the coordination numbers of Co–N and Co–Cu are 3.8 and 0.9, respectively, indicating that most Co atoms combine with adjacent Cu atoms to form CoN_4_–CuN_4_ dual atom pairs. On the other hand, EXAFS analysis and fitting results of Cu K-edge (Figure S6 and Table S1) confirm that the Cu atom in Co–Cu/PHI mainly bonds with a neighboring Co atom and four surrounding N atoms (the coordination numbers of Cu–N and Co–Cu are 4.1 and 1.0, respectively), while the Cu atom in Cu/PHI coordinates to 3.9 N atoms, conforming to a CuN_4_ single atom.

Therefore, all the available characterization data agree with the successful preparation of M/PHI by the molten salt method as single atoms in the case of Cu/PHI and Co/PHI and with the presence of neighboring CoN_4_–CuN_4_ dual atoms in the case of bimetallic Co–Cu/PHI. These results agree with those reports in the literature that have previously established the molten salt method as a general procedure for the preparation of single atom catalysts on PHI. As a novelty reported here, the measurements support the possible cooperation between Co and Cu due to their proximity in the bimetallic Co–Cu/PHI.^5,14^

### Photocatalytic Activity at Ambient Pressure

The series of M/PHI materials were tested as photocatalysts for the gas-phase CO_2_ hydrogenation at 300 °C. Four different pressures between *quasi-atmospheric* pressure or 24 bar initial pressure were tested in this study. Preliminary blank controls showed that at these two pressures no products were observed upon irradiation in the absence of either CO_2_ or photocatalyst implying that the two components, photocatalyst, and CO_2_, are necessary to observe product formation. Also, in the presence of both CO_2_ and photocatalyst, but in the dark, negligible CO_2_ conversion was observed. Thus, quantification of the dark thermal catalysis contribution to product formation under irradiation indicates that it is less than 5%, meaning that even though the reaction is carried out at 300 °C, the process is photocatalytic in essence.

Upon irradiation under atmospheric pressure, the evolution of CH_4_ and CO in various proportions as the only products was observed for the three photocatalysts Cu/PHI, Co/PHI, and Co–Cu/PHI. Figure 4 provides a comparison of the photocatalytic activity of the three materials at atmospheric pressure. As can be seen, Co–Cu/PHI is the most active photocatalyst of the series, producing CH_4_ and CO at almost identical reaction rates of 57 μmol h^–1^. The product evolution rates for each photocatalyst under the various conditions studied are presented in Table 2. It can be seen there that the product rate for Co–Cu/PHI is about 1 order of magnitude higher than the activity of Cu/PHI under the same conditions, in spite of having a similar, actually a little lower for Co–Cu/PHI, total metal loading of both photocatalysts. Co/PHI exhibited the lowest photocatalytic activity in the series, but this could be due to the lower metal content of this material. Comparison of the catalytic activity of Cu/PHI and Co–Cu/PHI illustrates the synergism of combining in one material both metal ions Cu^2+^ and Co^2+^, in comparison to M/PHI photocatalysts of a single metal. The origin of CO_2_ as the source of CH_4_ and CO was supported by performing isotopic labeling experiments with ^13^CO_2_, whereby the formation of ^13^CH_4_ and ^13^CO was confirmed by mass spectrometry (see Figure S9 in Supporting Information).

**Figure 4:**
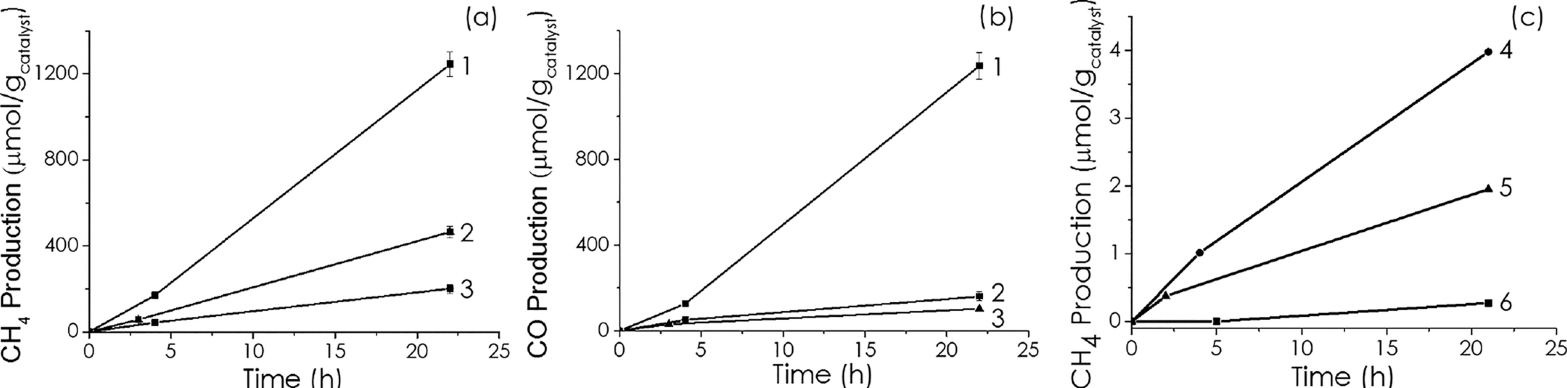
CH_4_ production (a), CO production (b) under UV–visible light at 300 °C using either Cu/PHI (plots 2 in graphs a and b), Co/PHI (plots 3 in graphs a and b), or Co–Cu/PHI (plots 1 in graphs a and b) as the photocatalyst. (c) CH_4_ production by filtering wavelengths shorter than the nominal value and in the dark from the output of the 300 W Xe lamp using Co–Cu/PHI as the photocatalyst. (1) Co–Cu/PHI, (2) Cu-PHI, (3) Co-PHI, (4) λ > 400 nm, (5) λ > 450 nm, and (6) dark conditions.

**Table 2:** Product Evolution Rates (μmol/*g*_catalyst_ × *h*) upon Irradiation in the Presence of Different Photocatalysts under Different Spectral Irradiation or in the Dark

	UV–visible light		λ > 400 nm	λ > 450 nm	dark
catalyst (pressure)	CH_4_ rate (mmol g^–1^ h^–1^)	CO rate (mmol g^–1^ h^–1^)	CH_4_ rate (mmol g^–1^ h^–1^		
Cu/PHI (1 bar)	21.1	4.7	-	-	-
Co/PHI (1 bar)	9.1	7.3	-	-	-
Co-Cu/PHI (1 bar)	56.6	56.2	2.0	0.2	0.01
Co–Cu/PHI (11.5 bar)	7.7	7.3			
Co–Cu/PHI (23.2 bar)	0.7	0.01			

To put the present results in a broader context, Table S2 in Supporting Information provides a summary of some of the reported results on photocatalytic CO_2_ using single atom catalysts including carbon nitrides that are compositionally related to PHI. However, the conditions of the reported studies are different, particularly, since most of them use H_2_O as proton and electron donor, while in the present work we are using H_2_ that has much lower oxidation potential hydrogenation. Not surprisingly, our results are better than those reported so far for single atoms. In contrast, in those precedents in which H_2_ was used as the reducing agent in the photocatalytic process, then, no single atoms have been used so far and our results are about twice higher than those achieved with nanoparticles, but at lower total metal loading (case of Co nanoparticles on hydroxyapatite in Table S2).

The photoresponse of Co–Cu/PHI was characterized by comparing at 300 °C the photocatalytic activity under the full UV–vis light output of the Xe lamp with that achieved in the near UV–vis and Vis regions using two different short-wavelength cutoff filters having a transmission window for wavelengths longer than 400 or 455 nm, respectively, and in the dark. The results are presented in Figure 4c. As it can be seen there, from the decrease in the amount of evolved CH_4_ and CO in comparison to the full spectrum irradiation upon correction for the lower light power, it can be deduced that a considerable percentage of about 55% of the total photoresponse is from the UV region from 200 to 400 nm. This is inferred from the evolution of CH_4_ and CO photoproducts, that decrease to less than one-half using the 400 nm cutoff filter and exhibit an even larger decrease to about only 30% of the total response on using the 455 nm filter. This performance is in good agreement with the UV–vis optical absorption spectrum of Co–Cu/PHI (Figure S4) which has a strong absorption in the UV, extending to the visible region, with an onset of about 460 nm. This absorption is characteristic of PHI and indicates that the photocatalytic process starts with photon absorption by PHI.

Light assistance at this temperature is clearly revealed by a comparison of product evolution under illumination with the dark reaction. Here, we found less than 1 order of magnitude lower CO_2_ conversion than the reaction under the full UV–vis light output at the same temperature. To further confirm that light is responsible for CO_2_ reduction, an additional experiment was carried out at 300 °C irradiation with a focused light intensity of about 70 sun power. Under these high light intensity conditions, the product selectivity drastically changed in favor of CO which exhibits a product selectivity of about 94.9%, with about 4.6% CH_4_ and detectable amounts of ethane. Interestingly, besides this change in product selectivity and the appearance of lower amounts of C_2_, the photocatalytic activity increased only by about 1 order of magnitude, reaching a CO production of 1389 μmol h^–1^.

The need for external heating in the photocatalytic CO_2_ hydrogenation is well documented in the literature and has been attributed to the poisoning effect of H_2_O for the active sites.^15^ Using the most active Co–Cu/PHI photocatalyst, no products were observed upon irradiation at ambient temperature or at 150 °C, while some CH_4_ and CO evolution was observed starting at 200 °C, the production rate under light irradiation increasing with the reactor temperature. A temperature of 300 °C was sufficient to perform the photocatalytic reactions. Most of the photocatalysts also exhibit activity as thermal catalysts for CO_2_ hydrogenations,^16^ but in dark conditions, the process requires much higher temperatures to occur at measurable rates. In the present case, the thermal catalytic activity of Co–Cu/PHI evolves at temperatures of 500 °C or higher, giving CH_4_ as the main product. As mentioned earlier, working at 300 °C under the present experimental conditions, the contribution of the dark thermal hydrogenation stayed always below 5% (see Figure 4c).

In order to gain insights into the mechanism of photocatalytic hydrogenation, a series of experiments using different electron donor quenchers in the absence of H_2_ gas and electron or acceptor agents were carried out.^17^ If light absorption by Co–Cu/PHI results in photoinduced charge separation with the generation of electrons and holes located at different sites on the material, then product formation from CO_2_ would correlate to the availability of electrons and protons. In this case, the presence of electron donors, such as aromatic compounds could accelerate the reaction in comparison to the use of molecular H_2_ as the hole quencher, depending on the oxidation potential of the sacrificial agent.^18^ Note that protons are then spontaneously generated from the primary organic radical cation formed by oxidation by the photocatalyst hole and there is no need to introduce hydrogen gas into the system. On the contrary, the presence of electron acceptors better than CO_2_ in the system would stop CO_2_ reduction, due to the preferential electron trapping by the acceptor in competition with CO_2_. Experimental photocatalytic data showed that, indeed, methane is formed from CO_2_ in the presence of anisole, thioanisole, and dimethylaniline, while the reaction is almost completely inhibited by the presence of nitrobenzene. The results are presented in Figure 5 and provide strong support for the operation of a photoinduced charge separation mechanism when using Co–Cu/PHI as a photocatalyst. The proposed mechanism is illustrated in Scheme 2. This mechanism would be in agreement with the well-reported photocatalytic activity of carbon nitrides.

**Figure 5:**
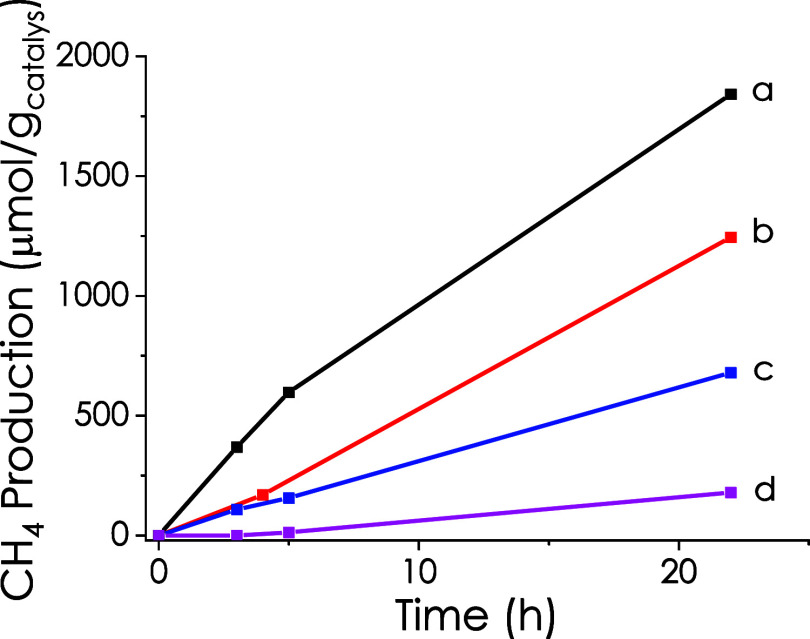
CH_4_ production using Co–Cu/PHI as a photocatalyst in the presence of quenchers: (a) Thioanisole + CO_2_, (b) H_2_ + CO_2_, (c) dimethylaniline + CO_2_, and (d) anisole + CO_2_.

**Scheme 2:**
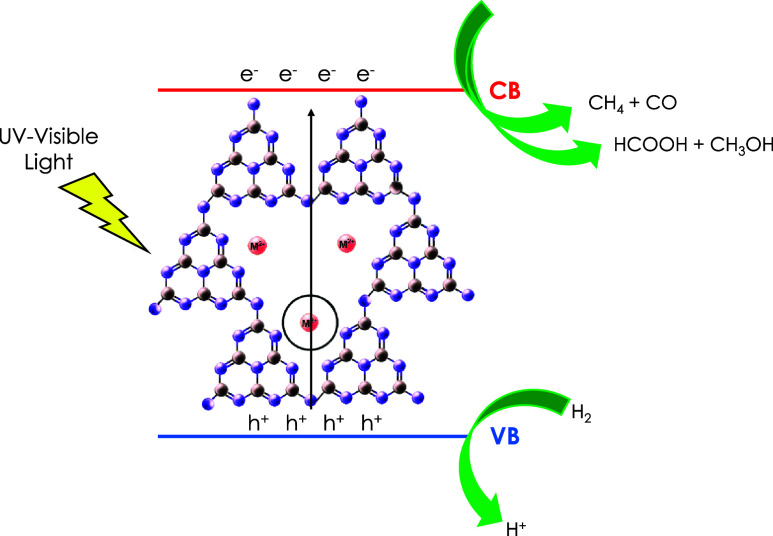
Proposed Mechanism Co–Cu/PHI as the Photocatalyst for the Photocatalytic CO_2_ Reduction Based on Photoinduced Charge Separation^a^

a Note again that usually only one or two of the possible three metal sites are occupied.

Further support for the operation of photoinduced charge separation with the generation of electrons and holes was obtained by comparison of XPS signals in the dark and under illumination. Monitoring the N 1s spectrum for Co/PHI a notable shift toward lower binding energy values of about 0.3 eV in the dark and upon irradiation was observed (see Figure 6). This shift toward lower binding energy was also recorded for the O 1s spectrum. Based on these shifts in the XPS binding energy values, indicating a higher electron density on the PHI N element, it is proposed that the photogenerated charge separation state corresponds to a metal-to-ligand electron transfer. Similar XPS studies were performed also for Cu/PHI and Co–Cu/PHI (Figure S10). However, in these cases, the shifts in the binding energy of the N 1s and O 1s peaks upon irradiation in the order of 0.1 eV were less clear than those observed for Co/PHI. In the literature, there are precedents in which these small shifts in XPS band maxima have also been observed and considered as evidence for the occurrence of photoinduced charge transfer.^19^

**Figure 6:**
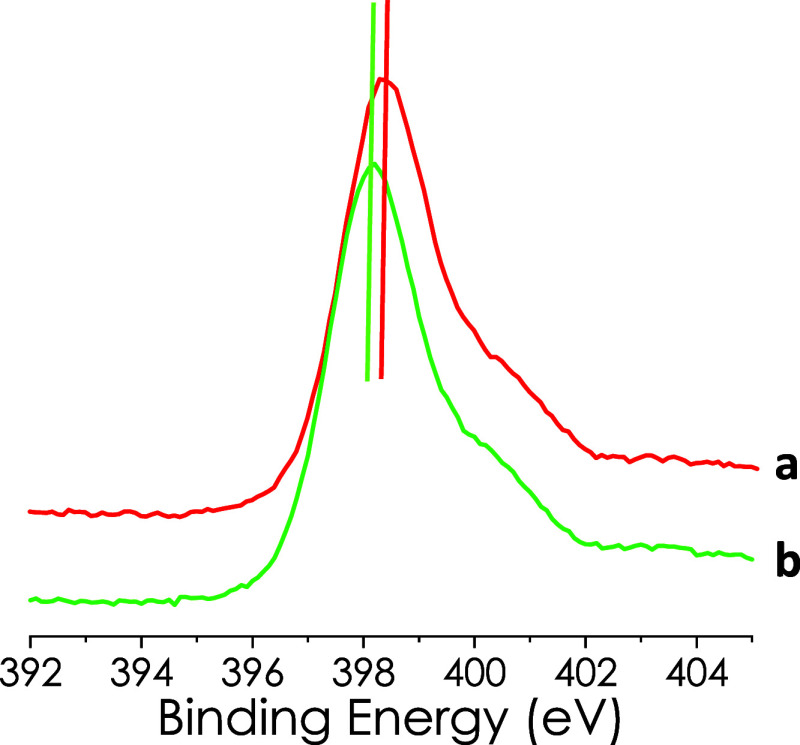
Comparison of the XPS N 1s peak for Co/PHI in the dark (a) and upon irradiation (b). The difference between the dark and light in the peak maximum is 0.3 eV.

In addition, the possibility of a photothermal mechanism in which the reaction is promoted by local temperature increase as the consequence of the conversion of the photon energy in heat at the nanoparticle seems less likely, as the reaction works also in the absence of H_2_ with alternative electron donor agents, such as anisole, dimethylamine, and thioanisole.

### Pressurized Photocatalytic Reactions

The previous photocatalytic experiments were carried out in the gas phase at about 1 bar of pressure. However, in thermal catalytic CO_2_ hydrogenations, it is known that pressure plays a key role in CO_2_ conversion and product selectivity.^20^ In order to determine if this pressure dependence also occurs in the photocatalytic reaction, while this can lead to a different product distribution, additional studies were carried out at 24 bar initial pressure and 300 °C. This is the maximum pressure that can be reached for a H_2_/CO_2_ 3:1 mixture with our pressurized isotopically labeled ^13^CO_2_ cylinder (6 bar maximum pressure). Note that since the photocatalytic reactor is charged at room temperature, due to subsequent heating of the system, the initial pressure at the time of charging the photoreactor increases during the reaction, reaching a maximum pressure value of 35 bar. The results of the photoirradiation were followed by analysis of the gas phase and the possible liquid products formed in the process, by washing the photocatalyst and the reactor with D_2_O aliquots that were subsequently analyzed by ^1^H and ^13^C NMR spectroscopy.

Under these conditions, upon photoirradiation in the presence of Cu/PHI formation of CH_4_ in the gas phase and formic acid and methanol in the D_2_O phase was observed at rates of 1.2, 13, and 23 μmol g^–1^ h^–1^, respectively. Formation of these liquid products from CO_2_ was firmly supported by ^13^C-labeling experiments, monitoring the reaction mixture by liquid ^1^H NMR spectroscopy in D_2_O solution, whereby observation of the ^13^C–H coupling, splitting the singlets corresponding to formic acid and methanol into doublets or doublets of doublets was recorded. Figure 7 presents some representative spectra to illustrate the ^1^H NMR spectra in solution from which the formation ^13^C-labeled formic acid (J_C–H_ 200 Hz) and methanol (J_C–H_ 134 Hz, J_H–H_ 12 Hz) was inferred. Importantly, these ^1^H NMR spectra also show the presence of some unlabeled formic acid and unlabeled methanol, which was estimated to be about 15% with respect to the total amount of methanol for the fresh photocatalyst. Control experiments using Co–Cu/PHI under 24 bar initial pressure and 300 °C as the photocatalytic CO_2_ reduction, but replacing CO_2_ by inert Ar, show the appearance at 3 h of minute amounts of formic acid and methanol, meaning that M/PHI undergoes some reaction under these conditions, releasing some formic acid and methanol of 3.0 and 1.5 μmol g^–1^ h^–1^, respectively, in substantially much lower amounts than the photocatalytic product when ^13^CO_2_ is present Thus, it seems that the origin of these unlabeled formic acid and methanol would be M/PHI or hardly avoidable carbon adsorbates on it. In any case, the ^1^H NMR spectra shown in Figure 7 clearly prove CO_2_ as the origin of most formic acid and methanol.

**Figure 7:**
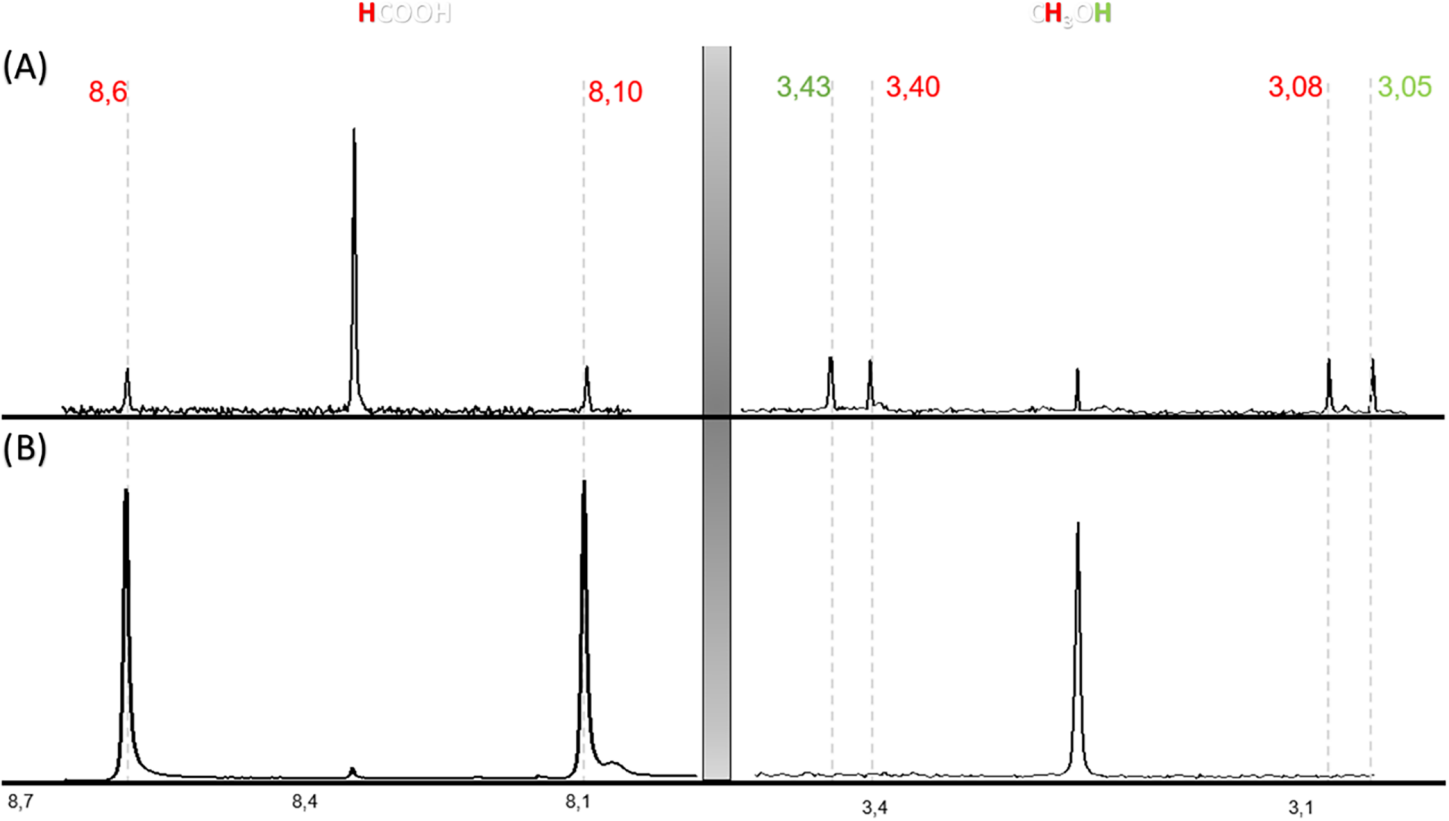
Expansion of the two relevant regions corresponding to formic acid (8.7–8.1 ppm, left side) and methanol (3.5–3.0 ppm, right side) of the 400 MHz ^1^H NMR spectra in D_2_O solution of the photoproducts resulting in the UV–vis irradiation of a 1:3 mixture of ^13^CO_2_ and H_2_ using Cu/PHI as the photocatalyst at 300 °C and 35 bar pressure recorded for the fresh (A) and five times used (B) Cu/PHI samples. Note that the signals at 8.37 and 3.25 ppm correspond to unlabeled H^12^COOH and ^12^CH_3_OH that are formed even when using ^13^CO_2_ as the carbon source, while the labeled H^13^COOH and ^13^CH_3_OH appear as doublet and doublet of doublets, respectively.

Upon subsequent use of the same Cu/PHI sample, a gradual significant decrease in the methanol formation in favor of formic acid was observed. Therefore, the gradual selectivity change would indicate that formic acid hydrogenation undergoes stronger deactivation in comparison to the formation of formic acid from CO_2_. Table 2 shows the reaction rates for the five consecutive uses of the same Cu/PHI sample. As indicated in eqs 1 and 2, the most likely origin of methanol is through formic acid hydrogenation. This was supported in an additional control experiment under identical experimental conditions at 24 bar initial H_2_ pressure, but using formic acid as the starting substrate instead of CO_2_, in which the formation of methanol was indeed observed.

1 $${\mathrm{CO}}_{2}+2e^{-}+2H^{+}\to \mathrm{HCOOH}$$

2 $$\mathrm{HCOOH}+2H_{2}\to {\mathrm{CH}}_{3}\mathrm{OH}$$

After the photocatalytic reaction, the used Co–Cu/PHI sample was characterized by XPS (Figure S11), steady-state photoluminescence (Figure S12), and time-resolved emission (Figure S13). XPS data of the used sample was coincident with that of the fresh sample, meaning that there are no changes in the oxidation state and coordination environment of C, N, and O. In contrast, photoluminescence shows an increase in emission intensity, indicating that unwanted electron–hole recombination has increased after the photocatalytic reaction. This is in agreement with the observed gradual deactivation due to the lower density of productive photoinduced charge separation. Also, the emission lifetime becomes shorter after the use of the material, again indicating a shortening of the charge separation lifetime. This deactivation of the photocatalytic activity can, therefore, be attributed to a lower efficiency of single metal atoms to trap charge carriers on PHI, which can be due to a change in their coordination sphere. These photophysical data are in agreement with the observed gradual deactivation of the M/PHI photocatalysts.

The need for two different sites for the photocatalytic formation of formic acid and methanol was somehow supported by the fact that in comparison to Cu/PHI, Co/PHI can form formic acid from CO_2_, but no methanol. Again, the lower photocatalytic activity of Co/PHI can be a reflection of its lower metal content. As in the ambient pressure photocatalytic measurements, the most active sample was the dual metal Co–Cu/PHI, although its performance with similar to that of Cu/PHI under these conditions. Table 3 also lists the product formation rates for formic acid and methanol by using Co/PHI and Co–Cu/PHI as photocatalysts. It could probably happen that high pressure favors adsorption on the metal sites to the point that the nature of the metal loses importance.

**Table 3:** 0Product Rates in Photocatalytic CO_2_ Hydrogenation under Various Pressures^a^

pressure (bar)	photocatalyst	reaction conditions	HCOOH rate (μmol g^–1^ h^–1^)	CH_3_OH rate (μmol g^–1^ h^–1^)
35	Cu/PHI	blank (no CO_2_)	3.0	1.5
	Cu/PHI^b^	1st use	13	23
	Cu/PHI	2nd use	18	21
	Cu/PHI	3rd use	25	12
	Cu/PHI	4th use	27	8
	Cu/PHI	5th use	31	4
	Co/PHI	1st use	20	-
	Co–Cu/PHI	1st use	45	15
11.5	Co–Cu/PHI^c^	1st use	--	--
23.2	Co–Cu/PHI	1st use	40	--

a Reaction conditions: temperature 300 °C, initial pressure 8, 16, or 24 bar, full light output from a 300 W Xe lamp, reactor volume 100 mL, reaction time 2 h.

b Some CH_4_ (rate 1.3 μmol g^–1^ h^–1^) is observed.

c the presence of ethylene (rate 49 μmol g^–1^ h^–1^) was observed.

As far as we know, the influence of pressure on photocatalytic CO_2_ hydrogenation has remained unexplored. To understand better the effect of pressure, additional experiments were carried out at pressures intermediate between ambient and maximum achievable pressure (24 bar at room temperature). Specifically, tests at 8 and 16 bar pressure at ambient temperature, resulting in 11.5 and 23.2 bar at 300 °C using Co–Cu/PHI were also carried out. The results are also included in Tables 2 and 3. As can be seen there, CH_4_ and CO formation undergoes a drastic change from 11.5 to 23.2 bar. At 23.2 bar, formic acid was the prevalent product formed with respect to methanol. In comparison, methanol formation was favored for fresh and first reuse of Cu/PHI at 35 bar.

### Understanding of the Co–Cu Synergy in Co–Cu/PHI

To gain some understanding of the reasons for the synergy between Co and Cu that could rationalize the better performance of Co–Cu/PHI in comparison to that of a single metal for photocatalytic CO_2_ hydrogenation, periodic DFT calculations were performed. As a model of the system, periodic rhombic 2D PHI-based nanosheets containing 48 C, 68 N, and 13 H atoms and the corresponding metal ion as single atom sites were constructed according to the literature.^21^ The model is presented in Figure 8a. The use of a large lateral unit cell should exclude interactions between adjacent metals due to the large separation distance.^22^ In order to minimize also the superfluous interactions between the periodic units, a vacuum layer of 20 Å along the *z*-axis was included in the periodic model.

**Figure 8:**
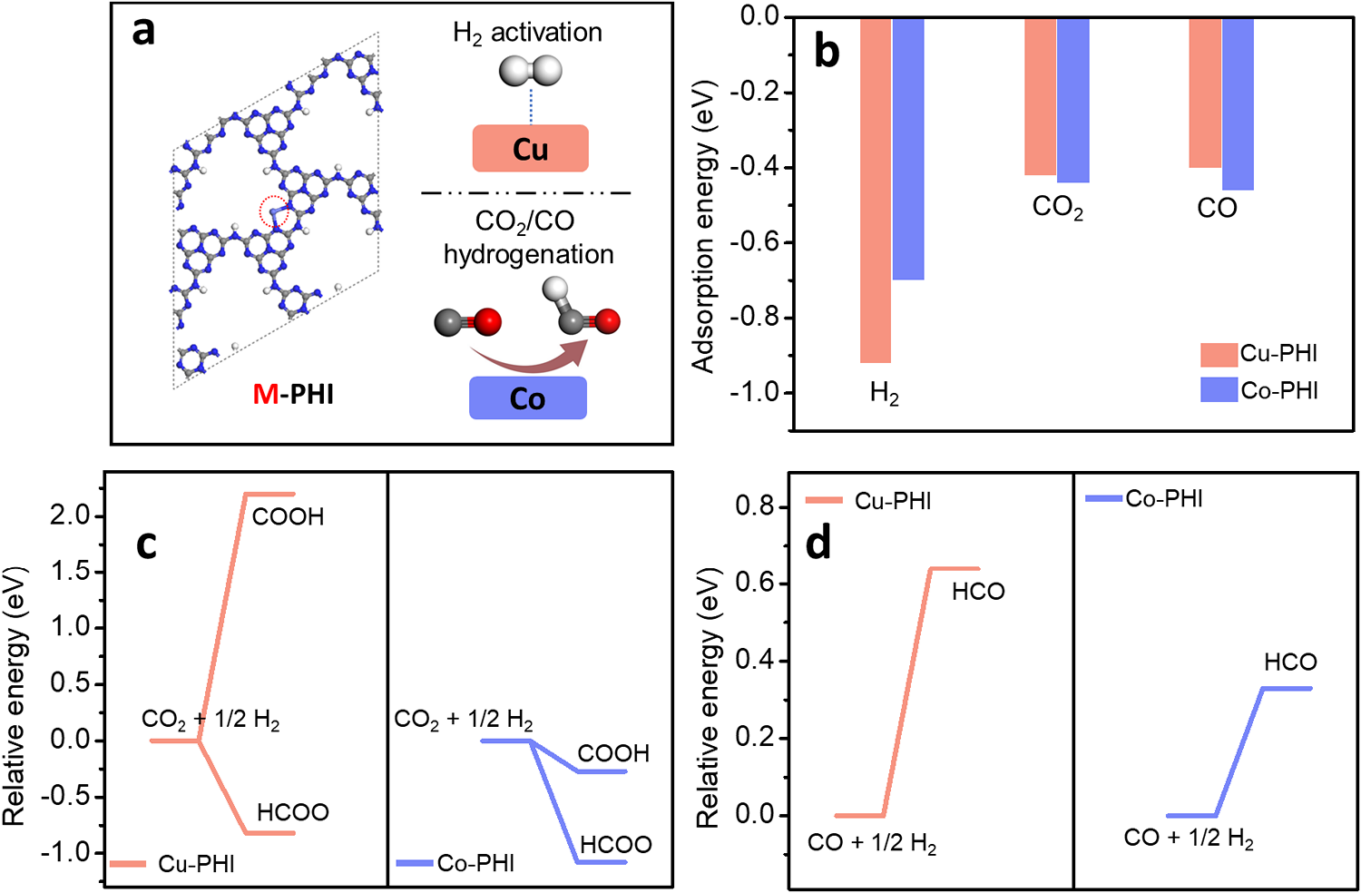
(a) Top view of the DFT-optimized configuration of the M/PHI structure (M = Co, Cu). The red circle in the model indicates the position of M. C and N atoms in gray and blue, respectively. (b) Adsorption energy of H_2_, CO_2_, and CO on M/PHI. Relative energy profiles of CO_2_ (c) and CO (d) hydrogenation on M/PHI.

To address the Co–Cu synergy, the adsorption energies of H_2_, CO_2_, and CO on the Cu/PHI and Co/PHI models were first investigated. The results revealed that H_2_ exhibits notably larger adsorption energy on Cu/PHI compared to Co/PHI, indicating an easier H_2_ activation at Cu atoms with respect to Co. Thus, activation of H_2_ over a single Co atom appears to be much less favorable than that on Cu. Conversely, the adsorption energies of CO_2_ and CO on both Cu/PHI and Co/PHI did not display significant disparities, with Co/PHI exhibiting slightly stronger adsorption energy values. The calculated adsorption energy values for Cu/PHI and Co/PHI are presented in Figure 8.

Then, the catalytic step involving the hydrogenation of CO* to HCO* was examined for both models. It was observed that the energy potential difference on Cu/PHI (0.64 eV) is notably larger than that on Co/PHI (0.33 eV), indicating that the Co sites are more favorable for hydrogenation of CO* to HCO*. These calculations are also indicated in Figure 8c. Therefore, due to the stronger CO adsorption and lower hydrogenation energy, single Co atoms are much better than Cu to catalyze the key step in the reaction mechanism.

In view of these calculations, it is proposed that the observed synergy between Co and Cu derives from the favorable situation occurring when the two metals are present in the system, i.e., on one hand, Cu activates H_2_ better than Co and on the other hand, Co promoting the hydrogenation of adsorbed CO to the key intermediate HCO* better than Cu. Therefore, the combination of the two in the proximity indicated by EXAFS would combine good H_2_ activation on Cu and fast hydrogenation of CO to HCO* on Co that would not occur with just one of the two metals.

## Conclusions

The data obtained have shown that single metal atoms on PHI can promote pressure-dependent photocatalytic CO_2_ hydrogenation. The photoresponse of Co–Cu/PHI derives mostly from the UV and blue parts of the visible region, in accordance with the optical absorption spectrum of PHI. Quenching experiments provide strong support to the occurrence of photoinduced charge separation as the primary event responsible for the photocatalytic activity, here CO_2_ reduction and hydrogen oxidation. The synergistic effect of the simultaneous presence of Cu and Co single atoms on PHI on the photocatalytic activity of CO_2_ hydrogenation is proven by comparing the activity of Co–Cu/PHI with that of the corresponding single atom Cu/PHI or Co/PHI photocatalyst at a similar total metal loading. Based on DFT calculations, we propose that this synergistic effect derives from Cu being better than Co to activate H_2_, while Co being better than Cu to hydrogenate CO into the key intermediate HCO*, thus, the combination of the two metals allows easy H_2_ activation due to the presence of Cu and a stronger CO adsorption and hydrogenation on the Co atoms. Pressure in the range from ambient to 35 bar exerts a remarkable influence on the photocatalytic reaction, changing the selectivity from gaseous to liquid products, giving rise to the formation of formic acid and methanol, as confirmed by the ^13^C-labeled CO_2_ experiments monitoring the product mixture by ^1^H NMR spectroscopy. The photocatalyst undergoes some side reactions under these conditions, affording also minor amounts of formic acid and methanol in the absence of CO_2_. The secondary product nature of methanol deriving from formic acid hydrogenation was supported by an independent experiment using formic acid as substrate as well as the gradual product distribution from CH_4_ to formic acid to CH_3_OH as the pressure increases. Worth noting is that liquid CO_2_-derived products, particularly methanol, have higher economic value than CH_4_ or CO, and their photocatalytic formation has resulted in controversial data in most cases.^23^ A progressive deactivation that results in a gradual diminution of methanol selectivity in favor of formic acid takes place but without undergoing a change in the structure of the PHI photocatalyst, poisoning of single atoms being the most likely deactivation pathway. Overall, the present study shows the potential that the combination of two different metals as single atoms can have due to the appearance of synergistic effects in the reaction mechanism derived from the cooperative contribution of each of them favoring certain steps in the reaction mechanism. This also provides a simple rationalization that opens the way for the *a priori* calculation of optimal metal combinations for CO_2_ reduction. In addition, the remarkable influence of the pressure favoring the formation of liquid products in the photocatalytic CO_2_ reduction has been disclosed offering new avenues for driving product selectivity away from economically less attractive CH_4_ and CO.

## Materials and Methods

### Preparation of M/PHI Samples

The M/PHI samples were prepared following a procedure previously reported with some minor adaptations to install Co, Cu, or Co–Cu metals.^24^ Briefly, a mixture of lithium and potassium chlorides 3 g each corresponding approximately to the eutectic proportion, together with 0.2 g of CoCl_2_ and CuCl_2_ or a 1-to-1 mixture of CuCl_2_ and CoCl_2_ and 2 g of 5-aminotetrazole were ground together in a mortar. The homogeneous mixtures were placed in an alumina crucible covered with a lid. The reaction was carried out in an electrical horizontal oven under constant nitrogen flow (150 mL min^–1^) and atmospheric pressure heating at a rate of 5 °C min^–1^ until 550 °C with a dwelling time of 4 h. After this time, the system was cooled at ambient temperature under N_2_ flow of the heating program, the crucibles were allowed to cool naturally to room temperature under a nitrogen flow, and the solid mixture was exhaustively washed with deionized water to remove soluble salts. M/PHI were filtered and dried in an oven at 60 °C overnight.

### Materials Characterization

X-ray diffraction (PXRD) spectra were acquired in the 2θ angle range between 2 and 90° at a scan rate of 10° per min with a Shimadzu XRD-7000 diffractometer using Cu Kα radiation (λ = 1.5418 Å), operating at 40 kV and 40 mA. Transmission electron microscopy (TEM) images were recorded using a Philips CM300 FEG microscope operating at 200 kV, equipped with an X-Max 80 energy dispersive X-ray detector (EDX) from Oxford Instruments. The electron microscope is equipped with the STEM unit and high-angle annular dark-field (HAADF) image detectors. TEM samples were prepared by dropping onto a carbon-coated Cu TEM holder a microdrop of the material previously dispersed in dichloromethane by sonication and allowing the dichloromethane to evaporate at room temperature before introducing it into the microscope chamber. Co and Cu analyses were measured by inductively coupled plasma-optical emission spectrophotometry (ICP-OES) with a Varian 715-ES analyzer. The samples were digested at 60 °C overnight with *aqua regia*, analyzing the mother liquid. ^1^H NMR spectra were recorded on a 400 MHz Bruker AV400 spectrometer using a known concentration of DMSO as the internal standard. XPS data were acquired on a SPECS spectrometer operating at 200 W equipped with a Phoibos 150 MCD-9 detector using a nonmonochromatic X-ray source (Al). Before spectrum acquisition, the samples were evacuated in the prechamber until an operating pressure of 1 × 10^–9^ mbar was reached. Quantification of the atomic ratios of the elements and spectrum analyses were carried out from the area of the corresponding peaks after background subtraction using a nonlinear Shirley-type correction and scaling the raw data according to the relative element response factor. The Co and Cu K-edge X-ray absorption fine structure (XAFS) spectra were acquired at BL11B beamline of Shanghai Synchrotron Radiation Facility (SSRF) of China. The working energy of the storage ring of SSRF was 3.5 GeV with an electron current in the top-up mode of 240 mA. The hard X-ray was monochromatized with a Si (111) double-crystal monochromator and the detuning was done by 30% to remove harmonics. The acquired EXAFS data were processed according to the standard procedures using the ATHENA module implemented in the IFEFFIT software packages.^25^ The *k* ^2^-weighted χ­(*k*) data in the *k*-space ranging from 2.5 to 11.0 Å^–1^ were Fourier transformed to real (*R*) space using Hanning windows (*dk* = 1.0 Å^–1^) to separate the EXAFS contributions from different coordination shells. To obtain the detailed structural parameters around Co and Cu atoms in the as-prepared samples, quantitative curve-fittings were carried out for the Fourier transformed *k* ^2^χ­(*k*) in the *R*-space using the ARTEMIS module of IFEFFIT.^26^

### Photocatalytic CO_2_ Reduction

Photocatalytic hydrogenation of CO_2_ at near atmospheric pressure was carried out in batch mode using cylindrical quartz photoreactors (51 mL) placed inside a heating mantle and a thermocouple to control the temperature. 25 mg of catalyst was introduced in the reactor and then, the reactor was purged initially with H_2_ and then the reactor was charged with a 3:1 H_2_/CO_2_ mixture. In a typical experiment, the photoreactor was heated at 300 °C, and then the photocatalyst was irradiated using a Xe lamp (150 W) that provided a collimated beam without or with short-wavelength cutoff filters of either >400 or >455 nm. The course of the reaction was followed by analysis of the head space with an airtight syringe injecting the mixture in an Agilent 490 MicroGC equipped with two channels and thermal conductivity detectors. To minimize artifacts due to excessive gas removal, sampling was made more often at short times to determine the initial reaction rates and, afterward, at final reaction times, taking four 1 mL sampling at most.

To study the reaction mechanism of the CO_2_ hydrogenation, blank controls were carried out under the same conditions in the absence of CO_2_, or using an Ar inert atmosphere. Other control experiments in the dark were carried out using the same quartz reactor (51 mL). Quenching experiments were performed using 25 mg of photocatalyst placed in the reactor, adding 20 μL of anisole, N,N-dimetilaniline or thioanisole, and then the reactor was purged only with CO_2_. The photoreactor was heated at 300 °C and the photocatalyst was irradiated using a Xe lamp (300 W). Analysis of the reaction products was carried out by injecting 250 μL aliquots with an airtight syringe in an Agilent 490 MicroGC equipped with two channels and thermal conductivity detectors.

Photocatalytic CO_2_ hydrogenation under pressure was carried out within a BE100-WT reaction kettle (Beijing Perfectlight Technology) of 100 mL volume capable of withstanding 5 MPa and 300 °C of temperature. First, the catalyst (50 mg) was loaded in a circular 10 mm stage on a 5 cm high Teflon support inside the reactor kettle. The photocatalyst was activated for 30 min at 300 °C with a 2 mL/min H_2_ flow followed by a 1 h cooling down under N_2_ flow in order to remove H_2_ before the reaction started. Once the photocatalyst activation was done, the reactor was filled with 24 bar of a 3-to-1 CO_2_/H_2_ mixture, and the reaction temperature was set. Once the reaction temperature was reached, the photocatalyst was irradiated through a transparent Zr window from the top by a 300 W Xe lamp during a 2 or 3 h reaction time, depending on the catalytic assay. The liquid and gas reaction products were analyzed. After cooling naturally the reactor in contact with the ambient, the gas phase was collected with an airtight Hamilton syringe and analyzed using a gas chromatograph (Agilent 7890 A GC System) equipped with a Carboxen–1010 PLOT capillary GC column and a TCD detector. He was used as a carrier gas. The liquid products were collected by washing the photocatalyst and the reactor body with D_2_O and analyzing the resulting solution by liquid ^1^H and ^13^C NMR spectroscopy with a Bruker AV400 (400 MHz) spectrometer using deuterated water as the solvent and a known amount of DMSO as internal standard.

### Computational Models and Methods

Periodic DFT calculations were conducted using the Cambridge Serial Total Energy Package (CASTEP) module with the exchange-correlation functional described by Perdew–Burke–Ernzerhof within the generalized gradient approximation (GGA-PBE).^27,28^ Tkatchenko and Scheffler (TS) dispersion correction scheme is incorporated along with the exchange and correlation functional to increase accuracy in the structural and vibrational properties.^29^ A self-consistent field method (tolerance 5.0 × 10^–7^ eV/atom) was employed in conjunction with plane-wave basis sets with a cutoff energy of 460 eV in reciprocal space. All structures are geometry-optimized until energy is converged to 5.0 × 10^–6^ eV/atom, maximum force to 0.01 eV/Å and maximum displacement to 5.0 × 10^–4^ Å. The adsorption energy of species on the catalyst surface was calculated as *E*_ads_ = *E*_total_ – *E*_A_ – *E*_sur_, where *E*_total_ represents the total energy of the catalytic surface with an adsorbed molecule, and *E*_A_ and *E*_sur_ are the energies of isolated adsorbate molecule and the clean surface, respectively. The energy of an isolated molecule (*E*_A_) is computed by placing it in the same lattice box.

## Supplementary Material



## Notes

Additional characterization data of fresh and used PHI samples and table with the EXAFs fitting data and literature survey on photocatalytic CO_2_ hydrogenation (PDF)
