# Supplementary material for: Synergistic Effect on the Photocatalytic CO2 Hydrogenation to Methanol Using Dual Co–Cu Single Atom Poly(heptazine imide): Influence of Pressure on Product Selectivity
Source: ACS Catal. 2025 May 21;15(11):9584–96. doi: 10.1021/acscatal.5c00827 (PMC12150263; doi:10.1021/acscatal.5c00827)
Supplement: Supplementary file 1 [file cs5c00827_si_001.pdf]

## Supporting information

**Synergistic effect on the photocatalytic $CO_2$ hydrogenation to methanol using dual Co-Cu single atom poly(heptazine imide). Influence of pressure on the product selectivity.**

Alberto García-Baldoví,[a] María Cabrero Antonino,[a] Lu Peng,[b] Liang Tian,[a] Sara Goberna-Ferrón[a], Germán Sastre,[a] Hermenegildo García,*[a] Markus Antonietti*[b] , and Ana Primo*[a]

[a] Instituto de Tecnología Química Universitat Politècnica de València-Consejo Superior de Investigaciones Científicas, Universitat Politècnica de Valencia, Av. De los Naranjos s/n, 46022 Valencia, Spain.

[b] Department of Colloid Chemistry, Max Planck Institute of Colloids and Interfaces, Am Mühlenberg 1, 14476 Potsdam, Germany.

E-mail addresses: hgarcia@qim.upv.es (HG), Markus.Antonietti@mpikg.mpg.de (MA), aprimoar@itq.upv.es (AP)

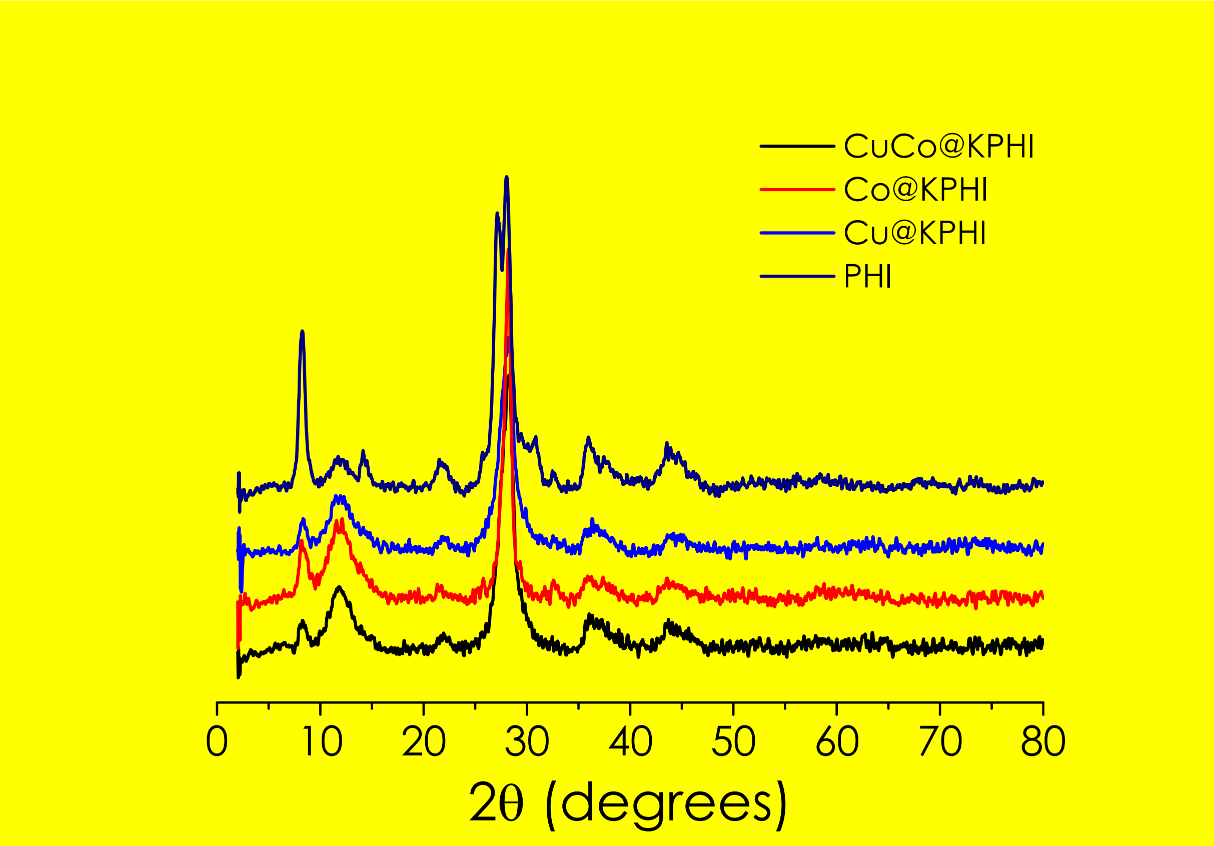


Figure S1. Powder XRD of the samples under study.

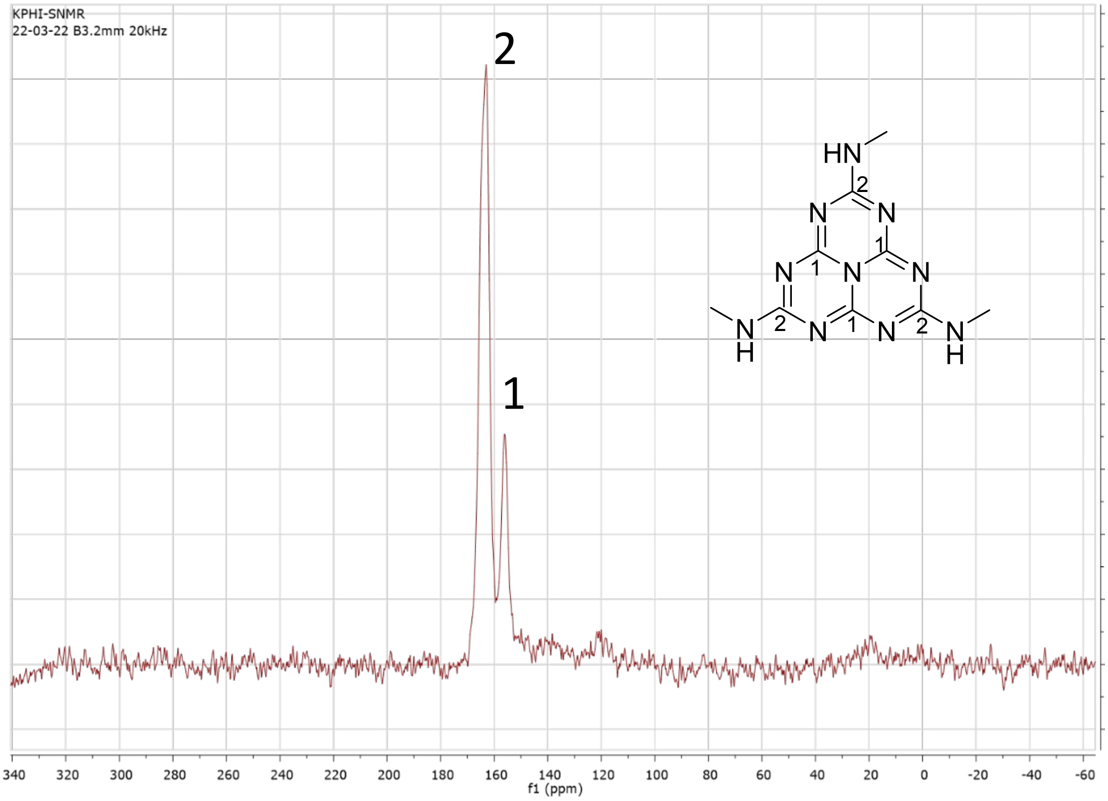


Figure S2. Solid-state magic angle spinning of $^{13}C$ NMR spectrum of KPHI.

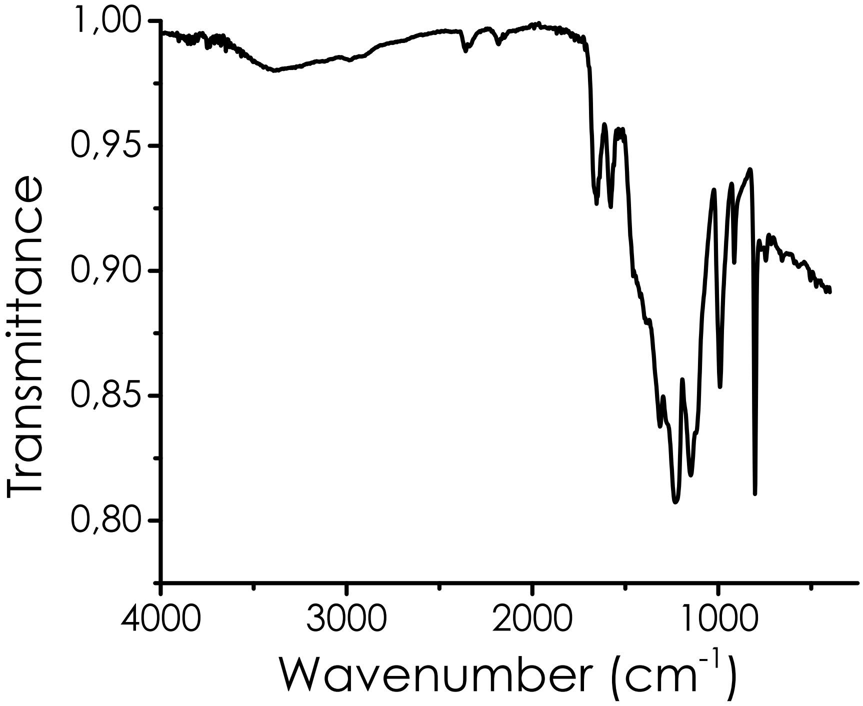


Figure S3. IR spectroscopy of KPHI.

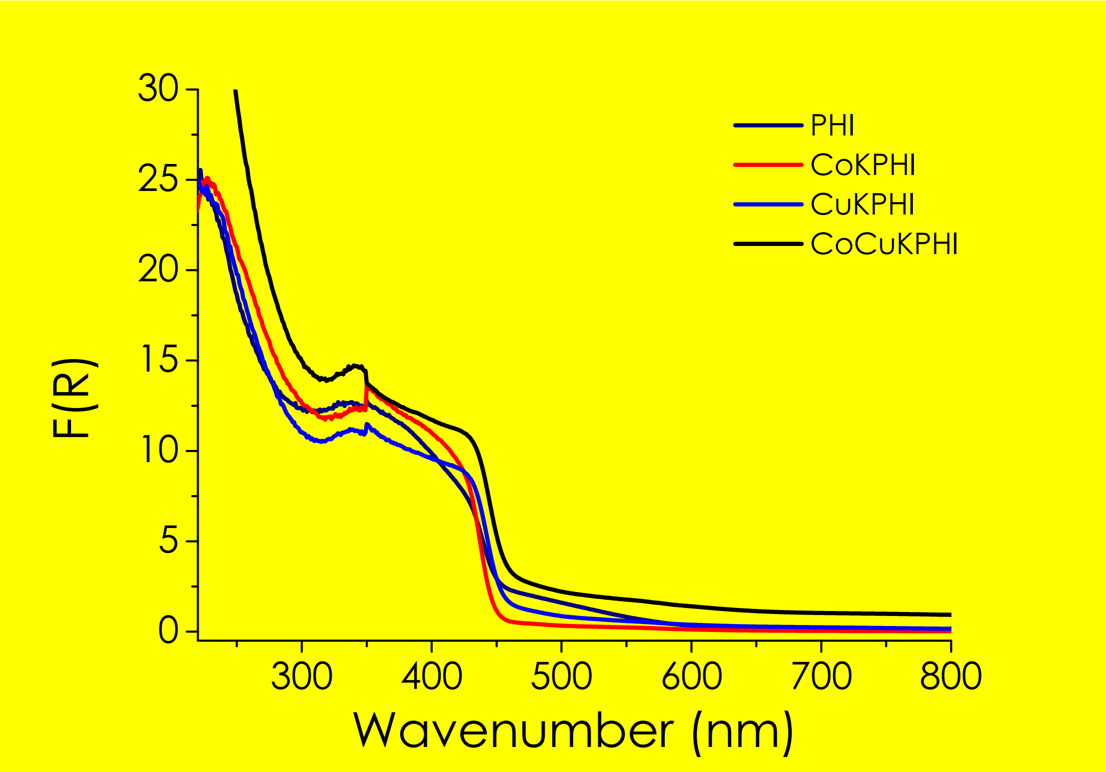


Figure S4. Diffuse reflectance of the samples under study.

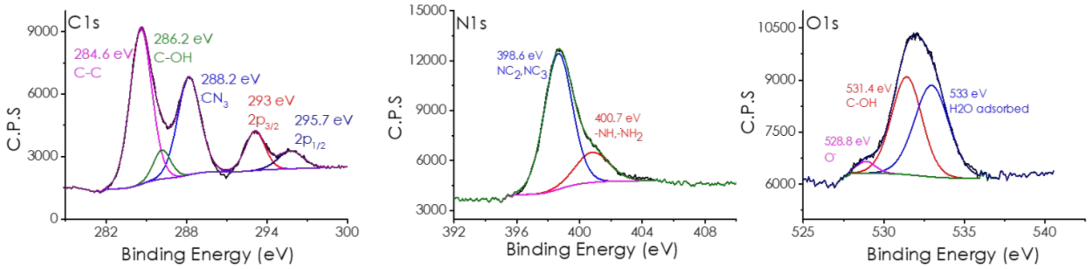


Figure S5. XPS spectra of Cu/PHI: (a) C1s, (b) N1s and (c) O1s.

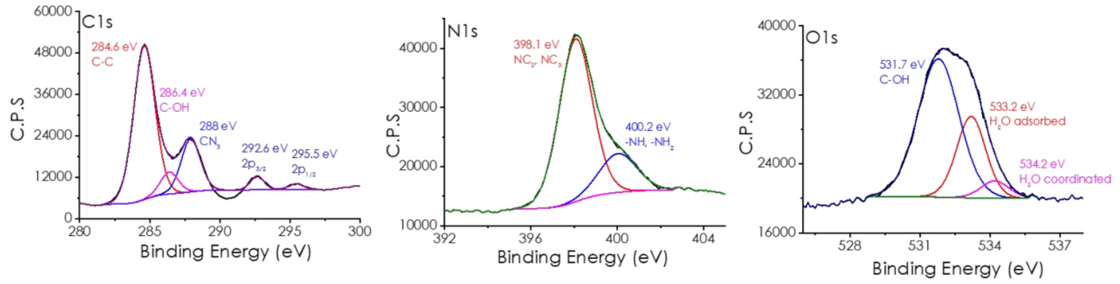


Figure S6. XPS spectra of Co/PHI: (a) C1s, (b) N1s and (c) O1s.

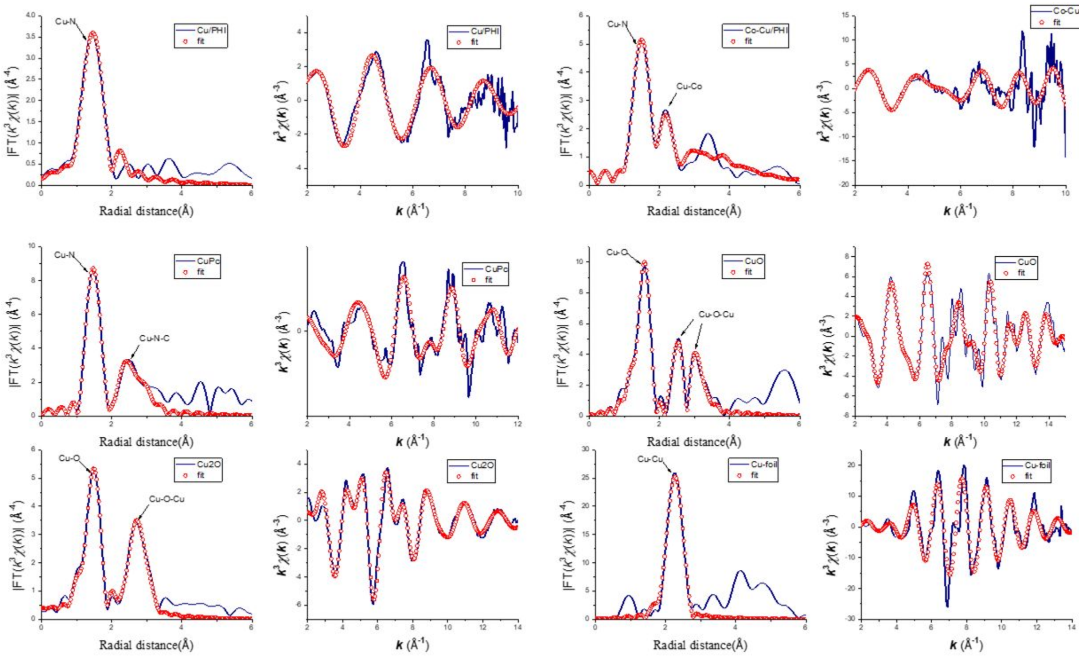


Figure S7. Cu K-edge fitting of the magnitude of the Fourier transform of the k3-weighted EXAFS (data-blue and fit-red) for Cu/PHI and Co-Cu/PHI samples and standards and the corresponding EXAFS at k space fitting curves of sample and standards.

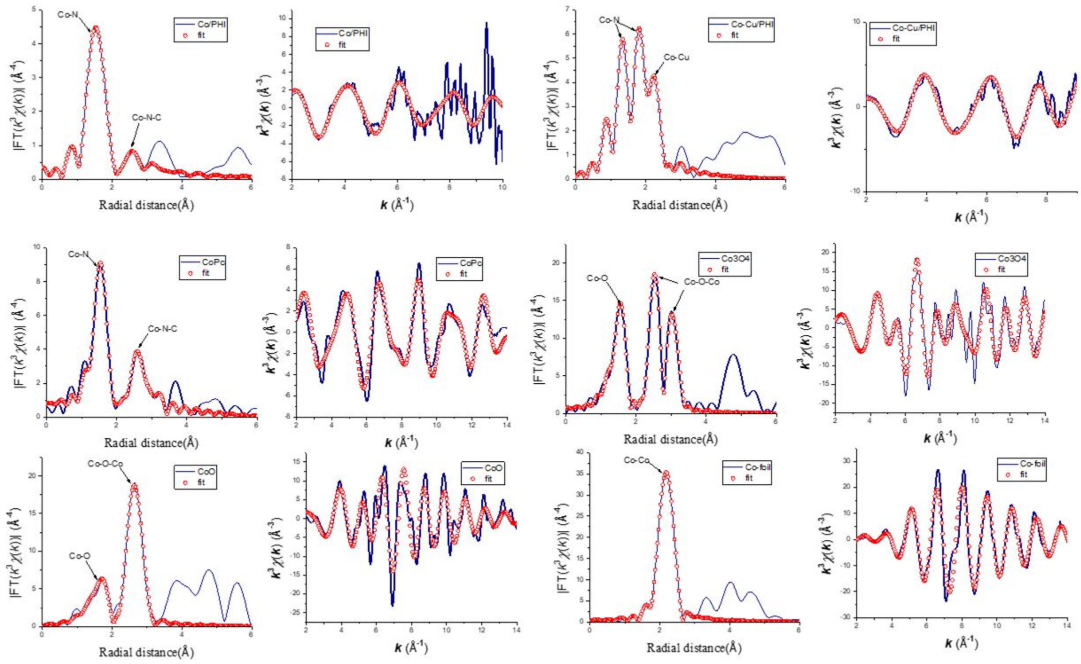


Figure S8. Co K-edge fitting of the magnitude of the Fourier transform of the k3-weighted EXAFS (data-blue and fit-red) for Co/PHI and Co-Cu/PHI samples and standards and the corresponding EXAFS at k space fitting curves of Co/PHI and Co-Cu/PHI samples and standards.

Table S1. EXAFS fitting data of M/PHI samples under study and standards.

| Sample | Path | $CN^a$ | $R$ (Å)$^b$ | $\sigma^2$ (Å$^2$)$^c$ | $\Delta E_0$(eV)$^d$ | $R$ factor |
|---|---|---|---|---|---|---|
| Co K-edge ($S_0^2$=0.835) | | | | | | |
| Co foil | Co-Co | 12* | 2.492±0.002 | 0.0061 | 7.7 | 0.0008 |
| CoO | Co-O | 6.0±0.1 | 2.126±0.023 | 0.0113 | 5.0 | 0.0073 |
| | Co-O-Co | 12.1±0.3 | 3.007±0.008 | 0.0087 | | |
| $Co_3O_4$ | Co-O | 6.0±0.2 | 1.916±0.005 | 0.0036 | 3.7 | 0.0051 |
| | Co-O-Co | 4.6±0.6 | 2.851±0.003 | 0.0035 | | |
| | Co-O-Co | 9.4±0.5 | 3.355±0.005 | 0.0065 | | |
| CoPc | Co-N | 4.0±0.1 | 1.918±0.009 | 0.0028 | 9.9 | 0.0043 |
| | Co-N-C | 7.2±0.3 | 2.943±0.018 | 0.0031 | | |
| Co/PHI | Co-N | 4.2±0.3 | 2.088±0.016 | 0.0030 | 7.6 | 0.0039 |
| | Co-N-C | 2.3±0.4 | 2.933±0.020 | 0.0083 | | |

| | | | | | | |
|---|---|---|---|---|---|---|
| Co-Cu/PHI | Co-N | 1.8±0.3 | 1.879±0.014 | 0.0037 | 4.1 | 0.0015 |
| | Co-N | 2.0±0.5 | 2.158±0.013 | 0.0038 | | |
| | Co-Cu | 0.9±0.2 | 2.546±0.020 | 0.0097 | | |
| Cu K-edge ($S_0^2$=0.872) | | | | | | |
| Cu foil | Cu-Cu | 12* | 2.551±0.007 | 0.0089 | 6.8 | 0.0086 |
| $Cu_2O$ | Cu-O | 2.0±0.1 | 1.867±0.007 | 0.0048 | 2.5 | 0.0055 |
| | Cu-O-Cu | 12.1±0.2 | 2.989±0.014 | 0.0150 | | |
| | Cu-O | 5.9±0.3 | 3.442±0.018 | 0.0041 | | |
| CuO | Cu-O | 4.0±0.2 | 1.954±0.004 | 0.0040 | 8.6 | 0.0093 |
| | Cu-O-Cu | 6.2±0.3 | 2.931±0.012 | 0.0097 | | |
| | Cu-O-Cu | 4.1±0.3 | 3.231±0.014 | 0.0110 | | |
| CuPc | Cu-N | 4.0±0.2 | 1.905±0.022 | 0.0041 | -7.3 | 0.0084 |
| | Cu-N-C | 6.8±0.4 | 2.948±0.016 | 0.0070 | | |
| Cu/PHI | Cu-N | 3.9±0.2 | 1.900±0.020 | 0.0093 | 6.4 | 0.0011 |
| Co-Cu/PHI | Cu-N | 4.1±0.3 | 1.906±0.018 | 0.0073 | -0.5 | 0.0073 |
| | Cu-Co | 1.0±0.2 | 2.543±0.013 | 0.0075 | | |

[a] *CN*, coordination number; [b]*R*, the distance between absorber and backscatter atoms; [c]$\sigma^2$, the Debye Waller factor value; [d]$\Delta E_0$, inner potential correction to account for the difference in the inner potential between the sample and the reference compound; *R* factor indicates the goodness of the fit. $S0^2$ was fixed to 0.835 and 0.872, according to the experimental EXAFS fit of Co foil and Cu foil by fixing *CN* as the known crystallographic value. * This value was fixed during EXAFS fitting, based on the known structure of Co and Cu. Fitting conditions: *k* range： 2.0 - 8.0; *R* range: 1.8-3.0 (Co/PHI); *k* range： 2.0 - 11.0; *R* range: 1.0-3.5 (Co-Cu/PHI) ; *k* range： 2.0 - 9.0; *R* range: 1.0-2.0 (Cu/PHI); *k* range： 2.0 - 12.0; *R* range: 1.3-3.5 (Co-Cu/PHI); fitting space: R space; *k*-weight = 3. A reasonable range of EXAFS fitting parameters: $0.800 < S_0^2 < 1.000$; $CN > 0$; $\sigma^2 > 0$ Å$^2$; $|\Delta E_0| < 15$ eV; *R* factor < 0.02.

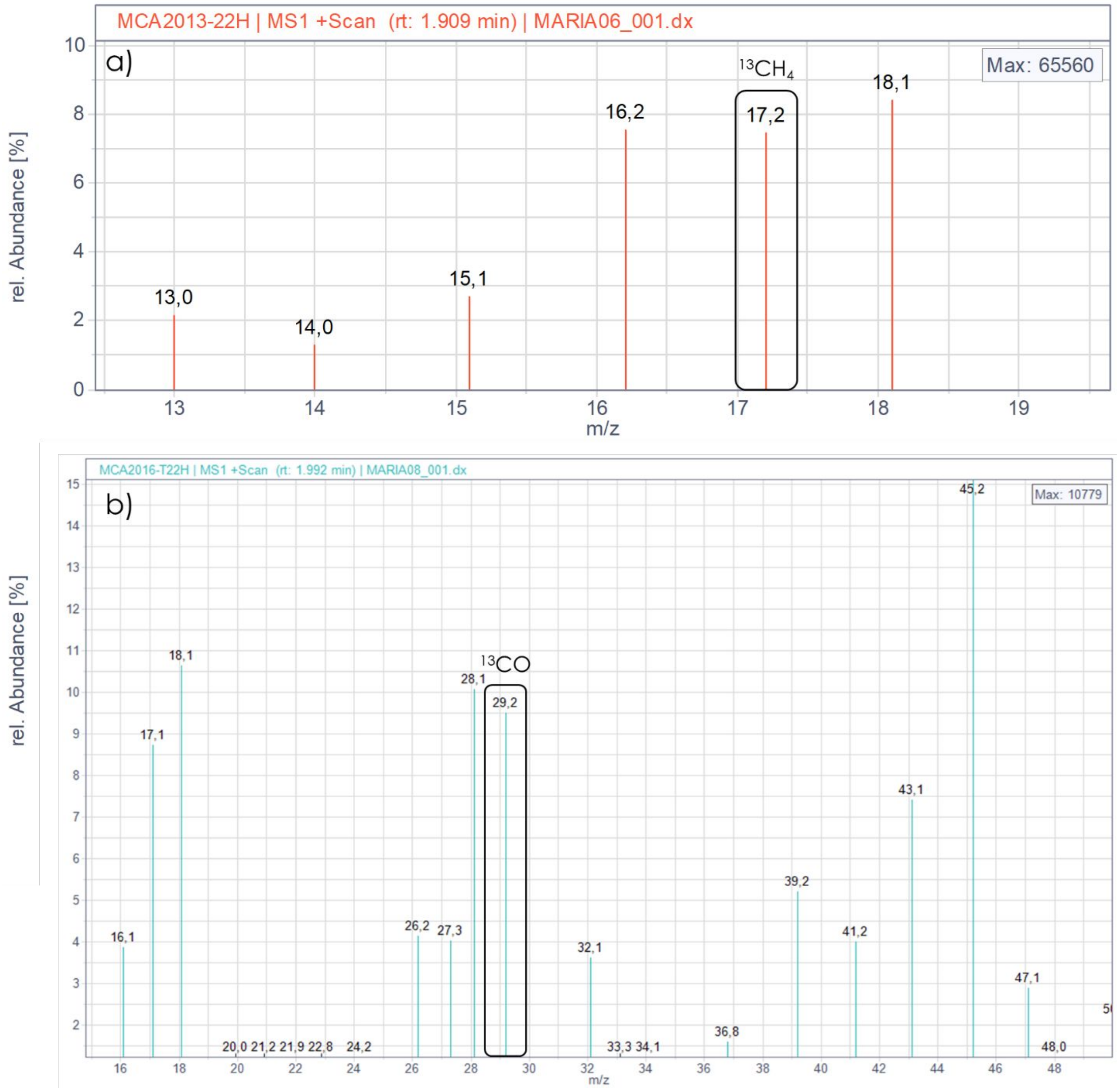


Figure S9. Mass spectra of the gas phase in the photocatalytic $CO_2$ hydrogenation at 300 $^{o}$C and atmospheric pressure using Cu/PHI as photocatalyst measured at 20 h irradiation time.

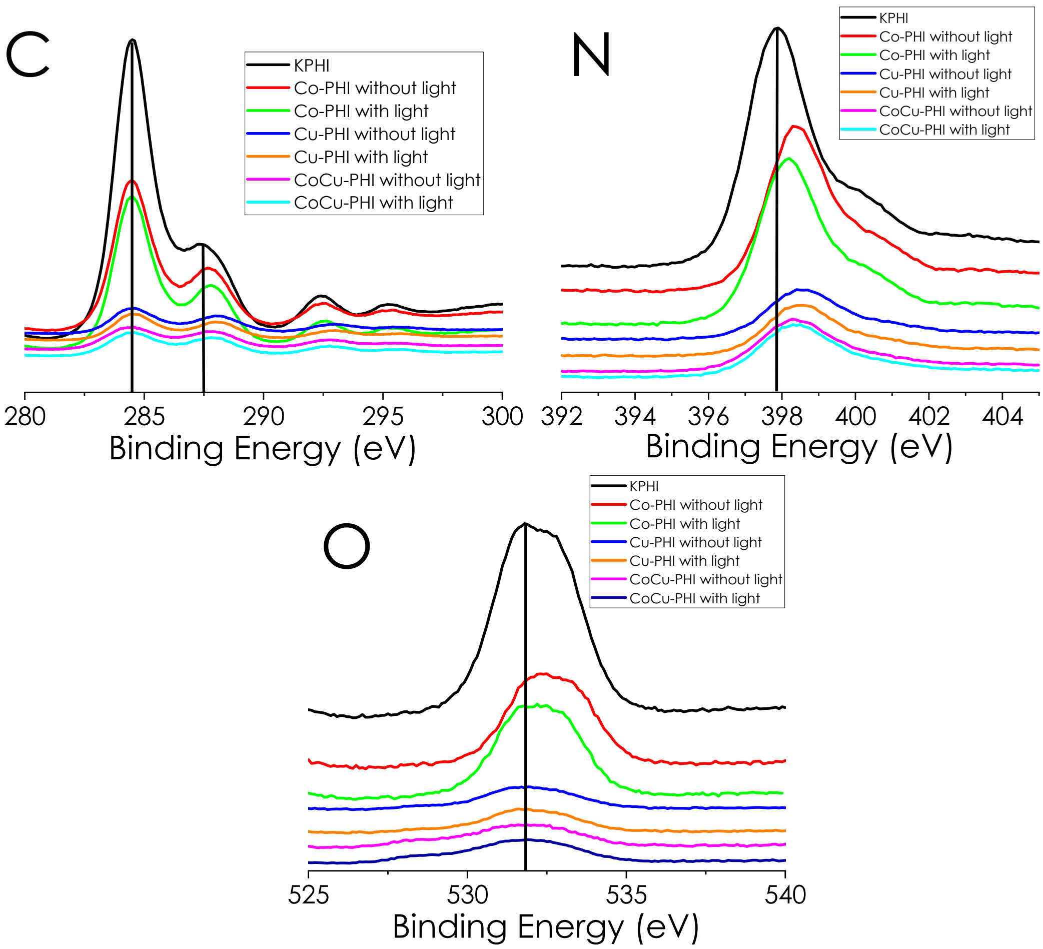


Figure S10. XPS comparison of the C1s, N1s and O1s for Co-PHI, Cu-PHI and CoCu-PHI in the dark and upon irradiation.

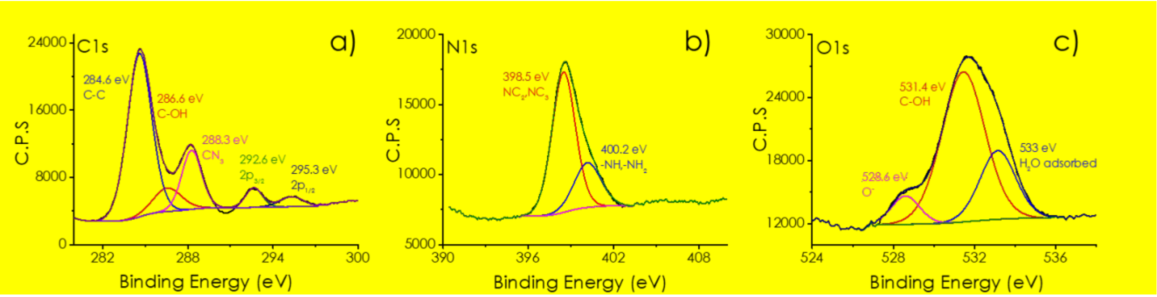


Figure S11. XPS data of reused Co-Cu/PHI; a) C1s, b) N1s and c) O1s.

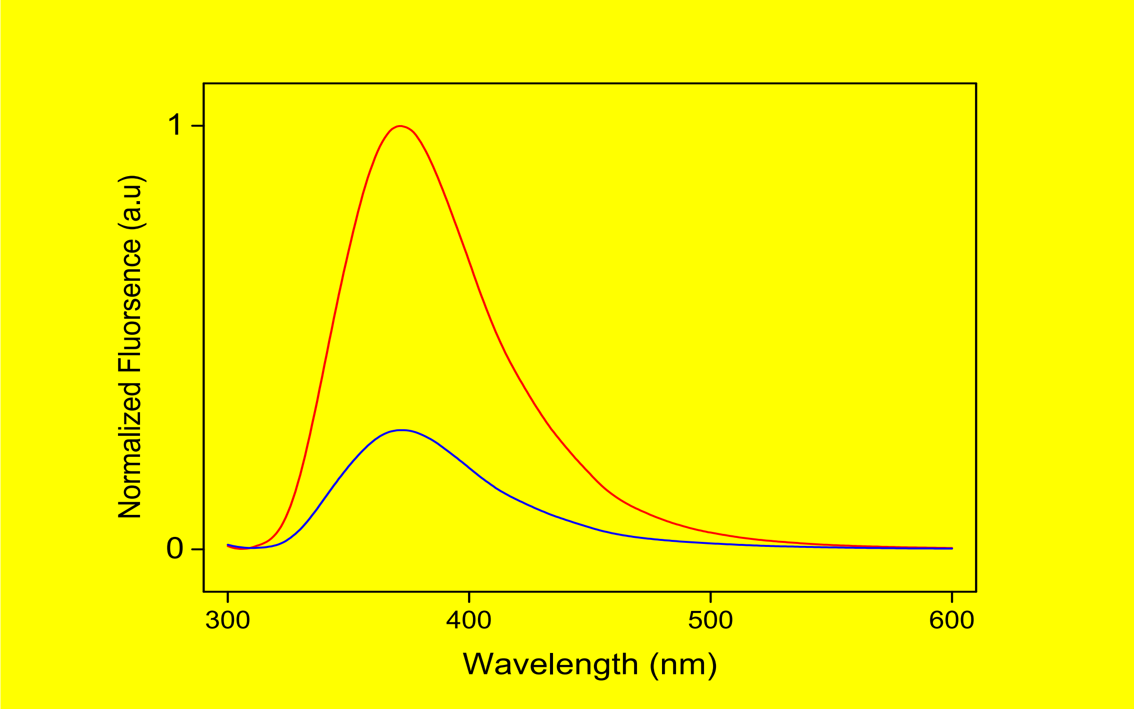


Fig. S12. Comparison of photoluminescence spectra for the fresh Co-Cu/PHI sample (blue) and after being used as photocatalyst for $CO_2$ hydrogenation under 35 bar pressure (red) measured in acetonitrile suspension (0.03 mg ml$^{-1}$) after $N_2$ purge upon excitation at 300 nm.

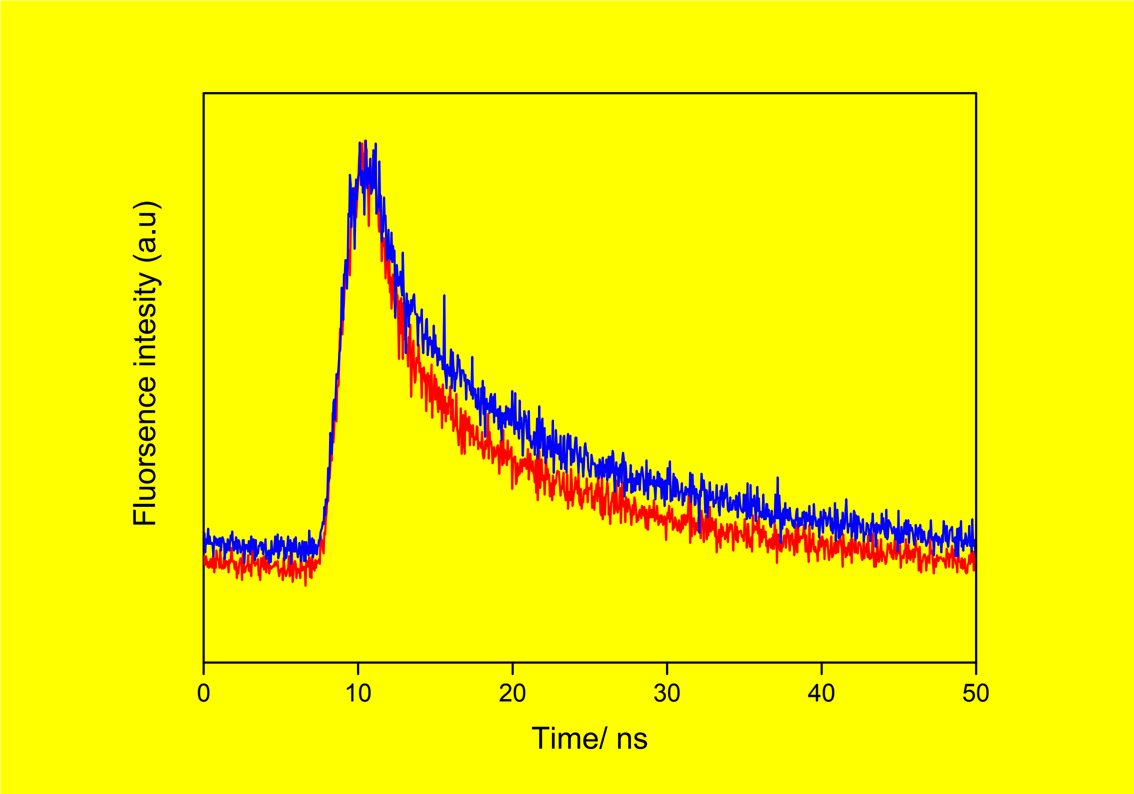


Fig. S13. Comparison of normalized emission decays for the fresh (blue) Co-Cu/PHI sample (blue) and after being used as photocatalyst for $CO_2$ hydrogenation under 35 bar pressure (red) measured in acetonitrile suspension (0.03 mg ml$^{-1}$) after $N_2$ purge upon excitation at 300 nm.

Table S2. Data from the literature reporting photocatalytic $CO_2$ hydrogenation on single atom catalysts.

| Photocatalyst | Product (formation rate) | Conditions | Ref. |
|---|---|---|---|
| Ag/$TiO_2$ | $CH_4$ (46.0 μmol g$^{-1}$ h$^{-1}$) | Solar light | [1] |
| Sn/$Co_3O_{4-x}$ | $CH_4$ (9 μmol g$^{-1}$) | Visible light | [2] |
| CoIn/carbon nitride | $CH_4$ (18.8 μmol g$^{-1}$) and CO (μmol g$^{-1}$) | 300 W Xe lamp, ambient temperature, $H_2O$ as source of protons and electrons | [3] |
| Cu (0.25%)/carbon nitride[a] | $CH_4$ (0.61 μmol g$^{-1}$), CO (11.21 μmol g$^{-1}$) and $CH_3OH$ (0.75 μmol g$^{-1}$) | 330 W Xe lamp though AM1.5 filter, $H_2O$ as sacrificial electron donor | [4] |
| B (1%)/carbon nitride | $CH_4$ (0.16 μmol g$^{-1}$) | 300 W Xe lamp. Gas phase using $H_2O$ as sacrificial electron donor | [5] |

| | | | |
|---|---|---|---|
| Co NP (8.5)/HAP [b] | CO (62 μmol $g^{-1}$) | 1 sun irradiation, 300 $^{o}C$, $H_2$ as sacrificial donor | [6] |
| Co-Cu/PHI | $CH_4$ (56.6 μmol $g^{-1}$) and CO (56.2 μmol $g^{-1}$) | 150 W Xe lamp, λ>400 nm, 300 $^{o}C$, $H_2$ as sacrificial donor | This work |

[a] The numbers in brackets correspond to the metal loading; [b] NP and HAP stand for nanoparticles and hydroxyapatite, respectively

## References


(1) Ban, C.; Wang, Y.; Feng, Y.; Zhu, Z.; Duan, Y.; Ma, J.; Zhang, X.; Liu, X.; Zhou, K.; Zou, H.; et al. Photochromic single atom Ag/TiO2 catalysts for selective CO2 reduction to CH4. *Energy & Environmental Science* **2024**, *17* (2), 518-530, 10.1039/D3EE02800C. DOI: 10.1039/D3EE02800C.

(2) Zhu, Y. Selective CH<sub>4</sub> production from CO<sub>2</sub> photoreduction via single-atom-promoted H<sub>2</sub>O dissociation. *Chem Catalysis* **2023**, *3* (10). DOI: 10.1016/j.checat.2023.100783 (acccessed 2025/03/27).

(3) Hu, B.; Li, Z.; Wang, B.; Chen, L.; Wang, X.; Hu, X.; Bai, Z.; Li, Y.; Chen, G.; Luo, X.; et al. Construction of Co-In dual single-atom catalysts for photocatalytic $CO_2$ reduction into CH4. *Applied Catalysis B: Environment and Energy* **2025**, *371*, 125196. DOI: https://doi.org/10.1016/j.apcatb.2025.125196.

(4) Wang, J.; Heil, T.; Zhu, B.; Tung, C.-W.; Yu, J.; Chen, H. M.; Antonietti, M.; Cao, S. A Single Cu-Center Containing Enzyme-Mimic Enabling Full Photosynthesis under CO2 Reduction. *ACS Nano* **2020**, *14* (7), 8584-8593. DOI: 10.1021/acsnano.0c02940.

(5) Fu, J.; Liu, K.; Jiang, K.; Li, H.; An, P.; Li, W.; Zhang, N.; Li, H.; Xu, X.; Zhou, H.; et al. Graphitic Carbon Nitride with Dopant Induced Charge Localization for Enhanced Photoreduction of CO2 to CH4. *Advanced Science* **2019**, *6* (18), 1900796. DOI: https://doi.org/10.1002/advs.201900796.

(6) Peng, Y.; Szalad, H.; Nikacevic, P.; Gorni, G.; Goberna, S.; Simonelli, L.; Albero, J.; López, N.; García, H. Co-doped hydroxyapatite as photothermal catalyst for selective CO2 hydrogenation. *Applied Catalysis B: Environment and Energy* **2023**, *333*, 122790.
